# Immunometabolism in biofilm infection: lessons from cancer

**DOI:** 10.1186/s10020-022-00435-2

**Published:** 2022-01-29

**Authors:** Rasoul Mirzaei, Niloofar Sabokroo, Yaghoub Ahmadyousefi, Hamid Motamedi, Sajad Karampoor

**Affiliations:** 1 Department of Microbiology, School of Medicine, Hamadan University of Medical Sciences, Hamadan, Iran; 2 Venom and Biotherapeutics Molecules Lab, Medical Biotechnology Department, Biotechnology Research Center, Pasteur Institute of Iran, Tehran, Iran; 3 Department of Microbiology, School of Medicine, Shahid Sadoughi University of Medical Sciences, Yazd, Iran; 4 Department of Medical Biotechnology, School of Advanced Medical Sciences and Technologies, Hamadan University of Medical Sciences, Hamadan, Iran; 5 Research Center for Molecular Medicine, School of Medicine, Hamadan University of Medical Sciences, Hamadan, Iran; 6 Department of Microbiology, School of Medicine, Kermanshah University of Medical Sciences, Kermanshah, Iran; 7 Gastrointestinal and Liver Diseases Research Center, Iran University of Medical Sciences, Tehran, Iran; 8 Department of Virology, School of Medicine, Iran University of Medical Sciences, Tehran, Iran

**Keywords:** Biofilm, Biofilm infection, Cancer, Metabolism, Immune polarization, Immunometabolism

## Abstract

**Background:**

Biofilm is a community of bacteria embedded in an extracellular matrix, which can colonize different human cells and tissues and subvert the host immune reactions by preventing immune detection and polarizing the immune reactions towards an anti-inflammatory state, promoting the persistence of biofilm-embedded bacteria in the host.

**Main body of the manuscript:**

It is now well established that the function of immune cells is ultimately mediated by cellular metabolism. The immune cells are stimulated to regulate their immune functions upon sensing danger signals. Recent studies have determined that immune cells often display distinct metabolic alterations that impair their immune responses when triggered. Such metabolic reprogramming and its physiological implications are well established in cancer situations. In bacterial infections, immuno-metabolic evaluations have primarily focused on macrophages and neutrophils in the planktonic growth mode.

**Conclusion:**

Based on differences in inflammatory reactions of macrophages and neutrophils in planktonic- versus biofilm-associated bacterial infections, studies must also consider the metabolic functions of immune cells against biofilm infections. The profound characterization of the metabolic and immune cell reactions could offer exciting novel targets for antibiofilm therapy.

## Background

Biofilm has been characterized as an accumulation of microorganisms embedded in extracellular polymeric substances (EPS) and attached to biotic or abiotic surfaces (Flemming et al. 2016, Mirzaei et al. 2020a; Mirzaei et al. 2020b). Biofilm has emergent attributes and novel properties that are not predictable from the survey of planktonic bacteria. Biofilm is one of the most broadly successful and distributed forms of life (Stoodley et al. 2002; Mahdiun et al. 2017). Bacterial biofilm is highly resistant to antibacterial drugs and host immune responses (Stoodley et al. 2002). It has been demonstrated that 80% of all bacterial infections in humans are associated with biofilm (Costerton et al. 1999; Mirzaei et al. 2020c).

The interplay among prokaryotes and eukaryotes is typical (Hakansson et al. 2018, 2022). This relationship affects the participants' metabolism on different paths, culminating in neutral, advantageous, and/or detrimental results for the members (Escoll and Buchrieser 2019). However, these fruitful interactions are less lasting and can potentially disrupt different agents, such as bacterial pathogens (Lupp et al. 2007). Metabolic changes in eukaryotic cells following different serious biofilm-associated infections are sometimes contradictory Mirzaei et al. (2020d). Frequent metabolic changes can occur in pathogens and host cells across biofilm-associated diseases (Molinero et al. 2019b).

On the other hand, the significant impact of tumor cells' metabolic changes and disease progression is well known, and metabolic reprogramming has been deemed one of cancer's hallmarks (Guo et al. 2019). The shift in main metabolic processes inside immune cells is now perceived to be due to nutrients or oxygen status and immune stimuli. It is evident that distinctive metabolic pathways, other than energy generation and biosynthetic pathways, control the shape and functions of immune cells (Guo et al. 2019; Mirzaei et al. 2021a; 2019). Physiological evaluations of bacterial infections showed that there is a close relationship between the host metabolic system and the host immune system (Belkaid and Hand 2014). This interaction begins soon after detecting pathogens and continues to evolve during their infection (Belkaid and Hand 2014). The current interest in the interface between host metabolism and host immunity has given rise to the field of ‘Immunometabolism’ (Guo et al. 2019, Mirzaei et al. 2021b). The host immune reaction generated in the biofilm infection is mostly ineffective, which results in chronic infections (Campoccia et al. 2019, 2020). This has been shown to occur through various pathways, which comprise direct encounters of neutrophils, macrophages, and myeloid-derived suppressor cells (MDSCs) (Rada 2015, 2017; Heim et al. 2014). Determining whether and how biofilm prevents host immune-mediated killing can help advance therapeutic approaches to enhance proinflammatory responses and facilitate the clearance of biofilm infections. Currently, it has known that alterations in the metabolism of immune cells can shape their function and phenotype (O’Neill and Pearce 2016; Loftus and Finlay 2016). For example, anti-inflammatory macrophages use oxidative phosphorylation (OxPhos), whereas pro-inflammatory macrophages favor aerobic glycolysis (Benoit and Koo 2016). This review will summarize the immune cells' metabolic reactions during biofilm infections compared with cancer.

## Biofilm and cancer: similarities and links

It has been documented that bacteria are encountered with various physiological states like anaerobic and dormancy based on the availability of micronutrients, pH, oxygenation, and bacterial metabolites generated inside biofilm (Maali et al. 2020). Following biofilm maturation, some aggregated bacteria could detach that, allowing colonization on other sites, resulting in the metastasis of the biofilm infection (Ivanenko 2021). Additionally, the extracellular milieu is a significant property that controls the function of bacterial and tumor cells alike. In this regard, for tumors, the extracellular matrix is usually changed and disorganized based on acidic pH and local hypoxia areas that impact the progression of the tumor (Petrova et al. 2018). Also, this extracellular matrix can be fibrotic and denser as tumors enlarge, resulting in limiting diffusion (Petrova et al. 2018). Taken together, the dysfunctional matrix causes diffusive barriers leading to micro-environmental heterogeneity that stops drug distribution (Ivanenko 2021). On the other hand, the microbial EPS matrix of biofilm also generates heterogeneities, localized pH, and oxygen gradients microenvironments, changing the bacterial survival and virulence (Koo et al. 2013).

The treatment of biofilm infections and solid tumors faces a similar problem; for example, drugs usually fail to reach and kill tumors and biofilm-embedded bacteria due to local micro-environment heterogeneities (Benoit and Koo 2016). Besides, traditional systemic and topical therapeutic approaches could be toxic cause significant damage to healthy tissues (Benoit and Koo 2016). For example, in cancer, low red blood cell, platelet, neutrophil, and immune cell counts are common side effects for chemotherapy, while ototoxicity, nephrotoxicity, and neuromuscular blockade are common in antimicrobials against biofilm (Ivanenko 2021). Also, treatments associated with biofilm, including administration of high-dose antibiotics, can cause substantial collateral damage such as microbiota dysbiosis, resulting in the proliferation of bacterial pathogens (Benoit and Koo 2016). Taken together, cancer and biofilm show several unique similarities that could be considered to design new therapeutic approaches (Ivanenko 2021; Benoit 2016). Most importantly, altered immune reactions are usually noted for biofilm and tumor microenvironments (Ivanenko 2021; Benoit 2016). Altogether, there are crucial challenges for traditional antibiofilm and anticancer drugs to overcome these situations where pathogens and cancer cells survive and cause the onset of disorders (Ivanenko 2021; Benoit 2016).

It has been found that the ability of biofilm-embedded bacteria to evade antibiotics is mainly because of persisters, resistance, and tolerance (Lebeaux et al. 2014). In this regard, persister and tolerant bacteria are noted for difficulties in biofilm-associated chronic infections (Yan and Bassler 2019). Also, the similar parallel between biofilm infections and cancer shows that anti-persister approaches could help administer cancer treatment (Ivanenko 2021). For example, the inhibition of lipid hydroperoxidas Glutathione Peroxidase (GPX4) prevents the persistence of cells and results in the prevention of drug resistance in cancers (Ivanenko 2021). Better characterization of the behavior of various cell states in microbial biofilm could cause a reduction in the ineffectiveness of current therapeutic choices for both biofilm infection and cancer. The overview of similarities in cancer and biofilm is depicted in Table 1.

**Table 1:** Overview of similarities and links between biofilm and cancer

Property	Cancer	Biofilm
Microenvironment	The tumor volume comprises a mixed population of cancerous cells and several local and invading host cells, secretory mediators, and extracellular matrix components, together defined as the tumor microenvironment (Yuan et al. 2016) The interplay of tumor cells with their environments determines whether the primary tumor is eliminated, metastasizes, or develops latent micrometastases, and these interactions have a significant impact on tumor growth (Yuan et al. 2016) In parts of the tumor that are proliferating, the surrounding arteries cannot keep up with the increased supply of oxygen, resulting in hypoxic zones inside the tumor and the tumor microenvironment. In order for hypoxia-inducible factors (HIFs) to be digested by the 26S proteasome, prolyl-hydroxylases must mark the HIFs before they are digested. Prolyl-hydroxylases are suppressed in hypoxic circumstances, resulting in the stabilization of HIFs, which in turn stimulates the expression of a variety of genes associated with tumor development and progression (Brassart-Pasco et al. 2020) The tumor microenvironment is comprised of several biomolecules, such as glycoproteins (fibronectin and laminin), collagens, proteoglycans, and polysaccharides, all of which have distinct physicochemical features (Brassart-Pasco et al. 2020)	A biofilm architecture that has been developed consists of microbial cells and a matrix. It is possible to find noncellular elements in the biofilm matrix as well, relying on the milieu in which the biofilm has evolved. Noncellular elements including mineral crystals, corrosion particles, clay or silt particles, or blood products could be discovered in the biofilm matrix (Donlan 2002) Notably, current data has shown that bacteria may absorb host components, including fibronectin, mucin, collagen, DNA, hyaluronan, and filamentous polymers, toward their matrix to build a denser biofilm (Walker et al. 2005, Alhede et al. 2020; Birkenhauer et al. 2014; Blanchette and Orihuela 2012) A steady gradient is established due to the development of the extracellular polymeric material matrix, which provides diverse localized environments on a small scale (Flemming et al. 2021) Throughout many situations, the biofilm matrix constitutes approximately 90 percent of the overall biofilm volume and is primarily constituted of lipids, polysaccharides, extracellular DNA (eDNA), and proteins, among other things
Genetic changes	Anomalous chromosomal numbers are common in cancer cells, and the DNA becomes progressively aberrant due to a plethora of mutations that occur inside them (Cavenee and White 1995) Some of these alterations are driver mutations, which means that they are fundamental for developing the cell into a malignant oneSeveral malignancies have changed the expression of genes and enzymes involved in DNA and histone modifications, altering the epigenomic landscape throughout tumor initiation and development (Chakravarthi et al. 2016) Investigations are finding the crucial regulatory functions performed by non-coding RNAs and non-coding elements of the genome during the development of a tumor and those involving protein-coding genes. Many of these genetic and epigenetic changes act in tandem to promote tumor growth and metastasis (Chakravarthi et al. 2016)	It has been hypothesized that the high cell density, enhanced genetic competence, and aggregation of mobile genetic elements that happen in biofilms offer an optimal mix of circumstances for successful horizontal gene transfer, including the absorption of resistance determinants (Flemming et al. 2016) It is significant to note that the behavior of an organism might influence the quantity and sources of horizontal gene transfer (HGT) that it receives from other organisms. When a bacterium is in a biofilm population, the frequencies of HGT are more significant than when the bacteria is in a planktonic setting (Madsen et al. 2012; Darmon and Leach 2014)
Drug resistance	Even while chemotherapy is initially effective against many kinds of cancer, resistance may develop these and other factors, such as metabolic alterations and DNA mutations that enhance drug resistance and degrading Several aspects of drug resistance in the tumor have been explored, including drug degradation, drug target modification, drug export, DNA damage repair, cell death suppression, and the epithelial-mesenchymal transition (Housman et al. 2014) The epigenetic alterations that might cause therapeutic resistance were also documented, and it has been hypothesized that such epigenetic variables may result in the establishment of cancer progenitor cells, which seem to be cells that do not die when traditional cancer medicines are administered (Housman et al. 2014)	A number of factors contribute to biofilms' increased antibiotic-tolerance, such as: (i) decreased antimicrobial dissemination or sequestering through the extracellular biofilm matrix; (ii) the occurrence of slow-growing and even latent cells ("persisters") that are highly resistant to antibiotics that address bacterial metabolism; and (iii) the transfer of genetic elements gene encodes resistance determinants as a result of close cell proximity (Flemming et al. 2016; Grassi et al. 2017; Stewart (2015))
Evading the Immune System	Tumor cells exert significant attempts to maintain the host's immune response at bay. This includes both the tumor cells themselves, which represent immunomodulatory surface molecules such as PDL1, B7, or human leukocyte antigen (HLA) G, less MHC1 or it is component -2 microglobulin (B2M), and the tumor's microenvironment, which is affected and exploited by the cancer cells (Muenst et al. 2016) Increased expression of regulatory T-cell populations and consequent anergy of cytotoxic T-cells, interaction with tumor-promoting macrophages (M2 macrophages), and upregulation of the immunosuppression enzyme indoleamine 2,3-dioxygenase (IDO) are all essential factors in the development of tumor (Selvan et al. 2016; Ward-Hartstonge 2017; Prendergast et al. 2017) TNF-α and TGF-β are secreted by both compartments, as are a variety of other substances like interleukins and interferon. These may both enhance tumor cell survivability on the one side and stimulate the milieu, especially the host immunity, in a pro-tumorigenic way depending on the circumstances (Lippitz 2013, 2018)	Overall, host immunity elicited by a biofilm infection is mainly unsuccessful, resulting in persistent disease. Many research revealed that this happens in many ways, including direct death of leukocytes—macrophages, MDSCs, and neutrophils—or immune reaction regulation (Hanke et al. 2013, Rada et al. 2017) As shown by the infiltration of MDSCs and macrophage polarization to the anti-inflammatory mode, it has been established that biofilm-derived compounds may effectively reduce pro-inflammatory responses Persistent biofilm diseases emerge from a failure to develop an efficient immune reaction, requiring physical separation and elimination of infected tissues/medical implants for therapy (Yamada and Kielian 2019)
Communication	Tumorigenicity is dependent on the ability of cells to communicate with one another in a healthful milieu. For years, most studies suggested that tumor cells were separated from their neighboring environment and that this lack of cell-to-cell interaction pointed to poor tissue equilibrium and tumor growth and progression. Nevertheless, multiple additional investigations have shown that cell-to-cell interactions were critical in altering the characteristics of the microenvironment in order to generate the tumor niche Eugenin (2019) The communication between cells, also called cross-talk within the tumor milieu, might be directly through cell-to-cell communication via gap junction crossing, electrical coupling, and adhesion molecules, or secondary by traditional paracrine signaling via cytokines, extracellular vesicles, and growth factors (Dominiak et al. 2020)	Among the most intriguing elements of the bacterial community, life is that it enables bacteria to interact utilizing chemical signals There is evidence that several of the chemical signals generated by cells and managed to pass through their outer membranes might well be perceived not only by members of the same cell species but further through distinct bacterial communities in the same biofilm population— and possibly even by more complicated organisms in certain instances (Percival 2011) Microorganisms may interact with one another through quorum sensing during the biofilm-building process. Quorum sensing governs the metabolic reactions of planktonic cells and may lead to the production of microbial biofilms and enhanced pathogenicity (Li and Tian 2012)
Stickiness	Healthy cells release chemicals that cause them to adhere to one another in a group. Tumor cells do not produce these chemicals and may "float away" to neighboring places or via the circulation or lymphatic system to distant parts of the body (Coman 1961)	Emerging data suggest that biofilm adherence is a significant contributor to biofilm-associated disorders in clinics and biofouling in industrial applications (2021) As more bacteria aggregate, they begin to produce sticky compounds known as extracellular polymeric components, which they use to encapsulate themselves
Appearance	Healthy cells and malignant cells might seem significantly distinct under a microscope. Compared to the normal cells, tumor cells can display substantially more variety in cell size—some are bigger than normal, and others are smaller than normal (2010) Furthermore, tumor cells frequently have an unusual structure, both in terms of the cell itself and the nucleus (which serves as the "brain" of the cancer cell). It looks that the nucleus is both bigger and darker than that of normal cells (Dey 2010)	Because microbial cells in biofilms are often in close interaction with one another, mechanical connections between surrounding cells are significant (Volfson et al. 2008) Mechanical instability caused by non—uniform proliferation commonly causes structural alterations throughout an organism's growth. A notable example is the development of 3D wrinkles in bacterial biofilms forming on soft surfaces, improving nutrition and signaling chemical accessibility (Fei et al. 2020)
Chronicity	As reported by World Health Organization, cancer has been one of the four most common chronic diseases in the world today (Pizzoli et al. 2019) Even though cancer can be carefully monitored and managed, it may not always be totally eradicated. It may manifest itself as a chronic (ongoing) condition, similar to diabetes or cardiovascular disease. When it comes to some cancer types, such as ovarian cancer, chronic leukemias, and certain lymphomas, this is often the case (Harley et al. 2012; Markman 2011)	Biofilms have long been recognized as the main cause of most chronic diseases, including osteomyelitis, rhinosinusitis, otitis, diabetic foot ulcers, chronic wounds in general, cystic fibrosis patients' chronic pneumonia, and implants, but are not restricted to these diseases (Kvich et al. 2020)
Metastasis (Spread)	One of the last stages of the tumor is metastasis Many tumor cells lose the adhesion molecules that promote stickiness and can disconnect and migrate via the circulation and lymphatic system to other human body parts (Wittekind and Neid 2005) Malignant cells enter the circulation or lymphatic system at this step and migrate to a different place in the body, wherein they continue to divide and establish the basis for additional tumors	Although a biofilm is frequently characterized as a "cozy home" in which inhabitant bacteria are sheltered from attack, bacteria may rupture their biofilm connections and escape to colonies other surroundings. This controlled process, known as biofilm dispersion, is found in a broad range of species and is initiated in response to some biological and environmental stimuli (Guilhen et al. 2017)
Treatment method	A hallmark of cancer therapy is the combination of two or more medicinal drugs that mainly target cancer-promoting or cell-sustaining processes (Bayat Mokhtari et al. 2017) Furthermore, combination treatment may be responsible for preventing deleterious impact on cell cells while causing cytotoxic activity on tumor cells. This may happen if one of the drugs in the combined program is cytotoxic to some other medication in normal cells, effectively preserving normal cells against cytotoxic activity (Blagosklonny 2005)	The traditional approach to treating microbial diseases is to tackle the causal pathogens explicitly; nevertheless, the development of biofilms increased the adequate levels of antibiotics to a considerably greater level (Jiang et al. 2020) Combination treatment is especially promising in the scenario of biofilms because the diverse composition of these microbial populations necessitates targeting cells in various metabolic stages (e.g., actively developing cells and latent cells) (Grassi et al. 2017)

**Fig. 1:**
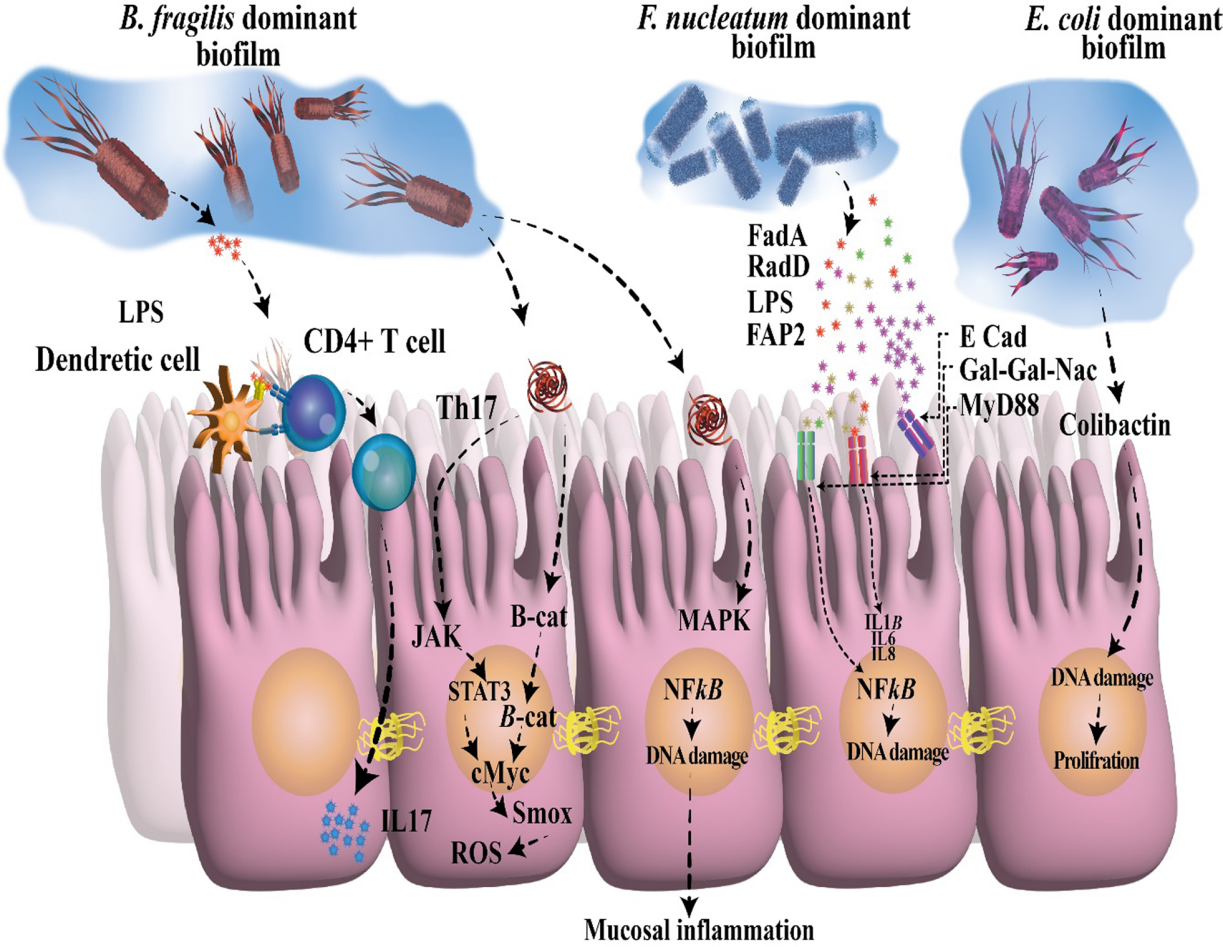
The polybacterial biofilm in colonic mucus. Bacterial biofilm can cause enhance the gut permeability, change of E-cadherin in colonic cells. Besides, biofilm can cause the loss of intestinal barrier activity, resulting in dysbiosis that could favor the enhanced growth of opportunistic pathogens. Eventually, the pro-oncogenic role of the biofilm and changes of polyamine metabolic and inflammation-associated Th17 prompt the growth of host cells, resulting in CRC initiation. CRC, colorectal cancer

Currently, some studies have found compositional changes in the tissue-mediated microorganisms in colorectal cancer individuals, for example, enterotoxigenic *Bacteroides fragilis* (ETBF), *Enterobacteriaceae* carrying the genotoxic polyketide synthase (pks), as well as *Fusobacterium nucleatum* (Ivanenko 2021). Nevertheless, the precise interplays between biofilm composition and cancer are being raised in this regard. *Enterobacteriaceae* and *B. fragilis* have been found inside mucosal biofilm from biopsies collected from individuals with inflammatory bowel disease (IBD), showing intestinal inflammation also impacts host susceptibility (Tomkovich et al. 2018). Fecal samples from individuals with carcinoma are enriched in *Parabacteroides*, *Bacteroides*, as well as *Escherichia* (Ivanenko 2021). Importantly, it has been found further link between biofilm-associated bacteria and colorectal cancer (Fig. 1) (Rizzato et al. 2019). In this regard, a study found polymicrobial biofilm production in colorectal mucosa of individuals with colorectal cancer (Rizzato et al. 2019). This phenomenon was striking for right-sided tumors compared to left-sided tumors (Dejea et al. 2014; Drewes et al. 2017). Interestingly, biofilm was present on tumors and at normal surgical margins. Besides, biofilm production was associated with reduced colonic epithelial cell E-cadherin, increased Interleukin-6 (IL-6) and Signal transducer and activator of transcription 3 (STAT3) stimulation, and enhanced crypt cell growth in normal colon mucosa (Dejea et al. 2014). Further analysis showed that these tumor-mediated biofilms were enriched with *B. fragilis*, *Peptostreptococcus stomatits,* and *F. nucleatum* (Rada 2017). Also, biofilm was found in colorectal mucosa of genetically predisposed patients, and it was rather patchy and primarily made of *B. fragilis* and polyketide-peptide genotoxin forming pks island positive *Escherichia coli* (Rizzato et al. 2019; Dejea et al. 2014). Additionally, it was also noted previously that *B. fragilis* is a crucial part of IBD-associated biofilm (Swidsinski et al. 2005).

Another bit of corroborative knowledge emerges from the increasing perception of the correlation between the oral microbiome, specifically *F. nucleatum*, and colorectal cancer, which was predominantly noted for proximal colon tumors (Sun and Kato 2016; Hussan et al. 2017). *F. nucleatum* is a routine residential part of dental plaques (biofilms) and prominent periodontal pathogen (Larsen and Fiehn 2017). *F. nucleatum* is deemed a co-aggregation expert, with an ability to co-aggregate with a wide range of bacteria, approximately all bacterial species implicated in oral plaque development, a crucial feature in biofilm formation (Kolenbrander et al. 1989; Allen-Vercoe et al. 2011). Besides, *F. nucleatum* can attach to and transport contrarily non-invasive bacterial species within host cells, working as a shuttle in this regard (Edwards et al. 2006). *F. nucleatum* constructs outer membrane vesicles (OMV) to promote co-aggregation, and separate OMVs only have been showed to exert an equivalent capacity to co-aggregate other bacteria examined to the complete bacterial culture (Kinder and Holt 1993). OMV generation seems to depend on external motives, and producing alterations in biofilm development are strain-specific (Martins et al. 2016). In this setting, the presumed correlation with colorectal cancer may perform the microbes that *F. nucleatum* associates in its biofilms rather than directly impacting its virulence. Flemer et al. (2018) recently described that various microbes regularly found in oral biofilms were enhanced in colonic mucosa from colorectal cancer cases. Ultimately, *F. nucleatum* has lately been discovered to be one of the gut microbes connected to pancreatic cancer, although limited known regarding biofilm production in the pancreatic ducts (Castillo et al. 2019). Further, investigations reveal that the commensal (such as *Parvimonas*, *Peptostreptococcus*, *Prevotella*) and the pathogenic (such as *F. nucleatum, P. gingivalis*) periodontal bacteria, which are able of creating biofilms, are identified in the intestinal biofilms (Drewes et al. 2017; Donelli et al. 2012). Hence, an intriguing hypothesis was intended to demonstrate the potential engagement of oral microbiota in colorectal cancer development. According to intestinal dysbiosis, the oral periodontopathic bacteria may have translocated within the colorectum. This introduces a fresh outlook on colorectal cancer pathogenesis which is operated by the orally-derived colonic biofilm (Koliarakis et al. 2019).

Intriguingly, a current murine investigation by Tomkovich et al. (2019), designed to portray the causality of microbial biofilms in colorectal cancer, strongly displayed that the polymicrobial biofilms are carcinogenic in a preclinical in vivo experimentation with the application of three murine genetic models of colorectal cancer carcinogenesis. An impressive conclusion of this research was that biofilm communities from the colon biopsies of healthy people were as robust as biofilm communities from colorectal cancer hosts in inducing the increase of tumors (Tomkovich et al. 2019). Moreover, the newest finding further determined that the same levels of inflammation were recognized in both mice treated with biofilm-positive and biofilm-negative control homogenates. Nevertheless, a lower grade of immunosuppressive myeloid cell recruitment and Interleukin-17 (IL-17) generation was triggered by biofilm-negative control homogenates when contrasted to biofilm-positive homogenates in the mice (Tomkovich et al. 2019).

### *Helicobacter pylori* biofilm and cancer

The gastric lumen is one of the common hostile circumstances in the human body, eliminating various bacteria in a few minutes. *Helicobacter pylori (H. pylori)* persist in this environment with its urease activity compensating gastric acid, whereas biofilm development may be further necessary for its persistent colonization (Abadi 2017). Surprisingly, concisely has been acknowledged concerning in vivo biofilm production on the human gastric mucosa to date. Three pioneer investigations operated a high-powered electron microscope and displayed thick clusters of *H. pylori*, originally in coccoid forms, which are identified as viable but non-cultivable (Carron et al. 2006; Coticchia et al. 2006; Cellini et al. 2008; Cellini 2014; Percival and Suleman 2014). It is fascinating to perceive that *H. pylori* isolated from gastric cancer cases are usually non-cultivable, also though the bacteria are detectable by distinct methods (i.e., polymerase chain reaction (PCR) or histology), and that coccoid forms have been certainly more commonly observed in gastric mucosa of gastric cancer cases than in that of peptic ulcer subjects (Chan et al. 1994). Indeed, *H. pylori* are the primary risk factor for the growth of gastric adenocarcinoma, which happens in almost 1 to 2% of infected people (Parkin 2006; McColl 2010).

Following its identification in the 1980s, investigations of *H. pylori *have concentrated on the planktonic, free-floating, nonattached form of growth; nevertheless, the current sign implies that *H. pylori* can also grow in a surface-attached biofilm form (Hathroubi et al. 2018). Investigations are starting to clarify *H. pylori* biofilm growth both in vitro and in vivo (Carron et al. 2006; Hathroubi et al. 2018; Servetas et al. 2016). While investigating *H. pylori* biofilms is almost new, the study in this unique field has gradually gained momentum over the past few years. Given the chronicity correlated with *H. pylori* infection, it is possibly not unexpected that this bacterium is recognized in biofilm (Hathroubi et al. 2018). Given that inflammation performs a crucial function in gastric carcinogenesis and that *H. pylori* have beneficial impacts on extra-gastric tissues, it may be therapeutically efficient to fine-tune the attendance of *H. pylori* in its active position in tissues where it is useful and target its inflammatory function in tissue where it is dangerous (Mentis et al. 2019, 2019). This approach could align with investigations revealing the anti-inflammatory performance of probiotics and other digestive factors such as Mastiha gum against *H. pylori* (Paraschos et al. 2007). Honey and yogurt eating have additionally been noted to be correlated with declines in *H. pylori* and anti-CagA Immunoglobulin G (IgG) seroprevalence, probably because of honey's antimicrobial activities and yogurt's probiotic and prebiotic actions (Yordanov and Boyanova 2017). It would be attractive to reveal if these results can be connected to biofilm activity, supposing that the quorum-sensing ability of biofilms can manage microbial community density.

The several procedures, including genomics, transcriptomics, and proteomics, are high-throughput that have been performed to recognize factors correlated with *H. pylori* biofilms (Hathroubi et al. 2018). One genomic investigation compared the sequences of wild-type strain J99 and 31 *H. pylori* clinical isolates that each had a spectrum of biofilm-forming capabilities as a method to distinguish genes enhanced in those strains that were high biofilm generators (Wong et al. 2016). The strains based on crystal violet staining intensity were categorized as low, moderate, or high biofilm generators (Wong et al. 2016). By analyzing the genetic diversity among strains, those contributors could identify several genes that seem to be correlated with biofilm production. Indeed, three hypothetical genes (K74_10375, K747_09130, and K747_06625) were remarkably associated with biofilm production (Wong et al. 2016). K747_06625 is prognosticated to hold a homing endonuclease and a ParB-like domain, the latter of which is correlated with biofilm development in some bacteria (Mashimo et al. 2013, 2014).

Additionally, four operative genes, coding for a flagellar protein (jhp_1117), an alpha-(1, 3)-fucosyltransferase, an outer membrane protein (OMP) (encoded by homD), and a cytotoxin-associated gene (Cag) pathogenicity island (PAI), were similarly correlated with biofilm development in *H. pylori* (Wong et al. 2016). The performance of the Cag PAI proteins in *H. pylori* biofilms was extra studied by the production of deletion mutations in CagA and the complete Cag PAI (Wong et al. 2016). Both mutations resulted in a notable reduction in biofilm biomass contrasted with that of the wild type (Wong et al. 2016). Additional work similarly advocated the significance of the Cag PAI proteins. Through employing a proteomic approach, Shao et al. (2013) recognized that two Cag proteins, Cag26/CagA and Cag24/CagD, were highly expressed in *H. pylori* biofilms provoked by serum deprivation related to planktonic *H. pylori* cultured below the same circumstances (Shao et al. 2013). Similarly, an investigation also revealed that the production of isogenic strains lacking CagE changed biofilm development by *H. pylori*, additional supporting the performance of the Cag PAI in biofilm production (Cole et al. 2004). These outcomes are especially relevant to pathogenesis because infections with Cag PAI-containing strains are usually correlated with an enhanced risk for gastric cancer (Nilsson et al. 2003).

## Host cellular metabolism during cancer and biofilm infection

Mammalian cells depend on basic metabolic processes for energy generation, macromolecule biosynthetic pathways precursors, and redox control power reduction (Vander Heiden 2009). Glucose, fatty acids, and amino acids (in particular glutamine) are the primary nutrients supporting these processes (Cantor and Sabatini 2012). Glucose is glycolytic metabolism's favored carbon energy source to provide acetyl-CoA for oxidation by the mitochondrial tricarboxylic acid (TCA) cycle (Martínez-Reyes and Chandel 2020). The pentose phosphate pathway (PPP) branching off glycolysis offers an alternate path for glucose degradation by producing nicotinamide adenine dinucleotide phosphate (NADPH) and ribose-5-phosphate for nucleic acid biosynthesis (Guo et al. 2019). As blood glucose levels decrease, the fatty acids from deposited fat are separated into acetyl-CoA through fatty acid β oxidation (FAO) in the mitochondrion (Schönfeld and Wojtczak 2016). This method is consistent with electron transfer to the electrons' transport chain to produce Adenosine Triphosphate (ATP) (Guo et al. 2019). The generated acetyl-CoA is applied to the TCA cycle or, more than the liver, recycled to form ketone bodies for extrahepatic tissues (Guo et al. 2019). Currently, it has been shown that FAO-formed acetyl-CoA enters the TCA cycle to form citrate, which can be conveyed to the cytoplasm to cause NADPH-generating isocitrate oxidation by isocitrate dehydrogenase (Carracedo et al. 2013, 2015; Williams and O’Neill 2018).

With the aid of novel cellular and molecular biological methods, cancer cell metabolism experiments have increased our knowledge of the pathways and functional effects of cancer-associated metabolic changes at different phases of carcinogenesis (Pavlova and Thompson 2016). Of note, it has become clear that tumorigenesis-mediated metabolic alterations cover all phases of cell-metabolite interplay, (1) determining the metabolic flux by conferring an enhanced capacity to obtain the required micronutrients; (2) changing the way micronutrients are selectively transferred to metabolic processes that lead to cellular tumorigenesis characteristics (3) and exercising long-term impact on cell destiny, including modifications in the divergence of tumor cells themselves and the parts of the microenvironment of cancer (Pavlova and Thompson 2016). A cell for providing the biogenesis conditions correlated with growth must enhance nutrition intake from the surroundings (Pavlova and Thompson 2016). The two primary nutrients that enable longevity and biosynthesis in cells are glutamine and glucose (Pavlova and Thompson 2016). Via the catabolism of glucose and glutamine, the cell retains reservoirs of diverse carbon intermediates that are employed as fundamental elements for constructing various macromolecules (Pavlova and Thompson 2016). Besides, regulated oxidation of glucose and glutamine carbon skeletons enables the cell to catch the reducing power each in the form of NADH and Flavin adenine dinucleotide (FAD) H2, which relate to the transition of electrons to the transport chain of electrons for the making of ATP energy or in the form of a relevant NADPH cofactor, which supplies reducing power for a broad range of biosynthetic re-engines (Pavlova and Thompson 2016).

The German physiologist Otto Warburg initially identified a dramatically elevated glucose absorption by cancers relative to non-proliferating normal tissues (Warburg 1924; Warburg et al. 1927). This finding has been verified in several tumor conditions and is associated with a bad tumor prognosis (Som et al. 1980). Positron emission tomography-based visualization of the absorption of a radioactive fluorine-labeled glucose relative, 18 F-fluorodeoxyglucose, has been applied to detect and stage tumors, as well as for testing exposure to care (Almuhaideb et al. 2011). Glutamine, the second main growth-supporting substrate, involves carbon and decreased nitrogen for de novo production of a variety of various nitrogen-containing substances (Pavlova and Thompson 2016). Glutamine provides the nitrogen demanded by the biogenesis of pyrimidine and purine nucleotides, glucosamine-6-phosphate, as well as non-essential amino acids (Pavlova and Thompson 2016). Also, glutamine has been known to play an activity in absorbing essential amino acids (Pavlova and Thompson 2016). While non-essential amino acids could be generated by mammalian de novo cells, essential amino acids must be obtained from external sources (Pavlova and Thompson 2016). It is essential to notice that the import of critical amino acid leucine via the plasma membrane-located neutral amino acid antidote L-Type Amino Acid Transporter (LAT1) was found to be combined with the concurrent efflux of glutamine (Nicklin et al. 2009). In this way, intracellular glutamine can promote importing a wide range of LAT1 substrates, such as valine, leucine, isoleucine, tyrosine, methionine, phenylalanine, and tryptophan (Yanagida et al. 2001).

Host metabolic activities are studied in various disciplines, including aging, immunity, and cancer. These include the catabolic biochemical processes for energy production (ATP) and anabolic reactions to produce biomolecules (Vander Heiden and DeBerardinis 2017; Finkel 2015; Escoll and Buchrieser 2018). There is a two-way relationship between the metabolic status and activity of individual host cells (Escoll and Buchrieser 2018). Hence, various metabolic pathways exist in proliferating, and non-proliferating cells and non-differentiated and differentiated cells, and these pathways are executed at a particular time. They are crucial for the functional status of cells (Escoll and Buchrieser 2018). Interactions between prokaryotes and eukaryotic cells are common and can affect the nutrients' metabolism in the environment, leading to beneficial, neutral, and harmful effects on the host cell. These interactions also occur between the host and its diverse microbiome (Mithieux 2018). These communications are short and could be altered by pathogens (Lupp et al. 2007). The metabolic communications that occur in eukaryotic cells during biofilm infection are competitive, in which the host cells try to limit nutrient access to the biofilm bacteria.

In contrast, the bacteria defend by taking advantage of the host micro-nutrients and metabolites (Molinero et al. 2019a). Metabolic changes in host cell following biofilm infections are of growing importance for understanding the pathogenesis of bacterial biofilm infections. A growing body of studies has been performed, mainly focused on the inflammation and immune reactions, to elucidate host responses in biofilm infections. For example, the formation of endosomal vesicles, trafficking, autophagy, host cell survival, and apoptosis have been studied, while the effects of biofilm infections on host metabolism are less studied (Huang and Brumell 2014; Lazar et al. 2018; Molinero et al. 2019a; Zhang et al. 2017). Most of the work on host reactions is mainly associated with signaling pathways and enzymes or transcription factors that influence the metabolism of nitrogen, carbon, and energy metabolism (Eisenreich et al. 2013, 2019).

Bacteria are increasingly being found to be active in stimulating and promoting cancer, especially colon cancer. Dejea et al. (Eisenreich et al. 2013, 2019) have characterized a biofilm description and its metabolic involvement in colon cancer in pioneering work. They found that biofilm can alter the mode of epithelial cells concerning oncogenic progression including decreased tumor suppressors, increased levels of inflammatory and angiogenic cytokines decrease in E-cadherin, and activation of IL-6, and STAT3 (Dejea et al. 2014; Dejea and Sears 2016). The colon biofilm as a pathogenic form has previously been associated with Crohn disease, IBD, and ulcerative colitis. Dejea et al. (Dejea et al. 2014; Dejea and Sears 2016) discovered that biofilms from colon cancer alter epithelial cell proliferation and metabolism in this context. It has been shown that in colon cancer, the rate of N (1), N (12)-diacetylspermine was increased in response to biofilm (Dejea et al. 2014; Dejea and Sears 2016). Higher levels of different polyamines have been diagnosed in colon cancer where the molecules of N (1), N (12)-diacetylspermine were found in the urine of patients with colon cancer, suggesting direct linkage of polyamine metabolites and biofilm formation (Dejea et al. 2014; Dejea and Sears 2016). The idea that biofilm can influence different metabolite molecules that affect the carcinogenesis process is a new area that needs further research (Hiramatsu et al. 2005). The recognition that biofilm communities can control the risk of oncogenesis is a starting point for new lines of research that can provide great insight into the role of the microbiome in colon cancer.

## Immune polarization

In vitro investigations of macrophage activation resulted in the discovery of M1 (classical) and M2 (alternative) modes for macrophages, which promoted the idea of immunological polarization. These states characterize macrophage proinflammatory vs anti-inflammatory properties, respectively (Martinez and Gordon 2014, Karampoor et al. 2021). On the other hand, it is well-found that in vivo, macrophage activation occurs along with pro-inflammatory and anti-inflammatory conditions, with a degree of flexibility in both directions (Yamada and Kielian 2019). Immune cell polarization is widely detected in host responses related to inflammation, cancer, microbial immunity, fibrosis, and tissue regeneration (Ma 2020). Immune cells develop diverse programming and execute specialized roles in response to particular signals throughout this process (Ma 2020). This section will overview the polarization of immune responses in the context of cancer and biofilm infection.

### Immune polarization in cancer

**Fig. 2:**
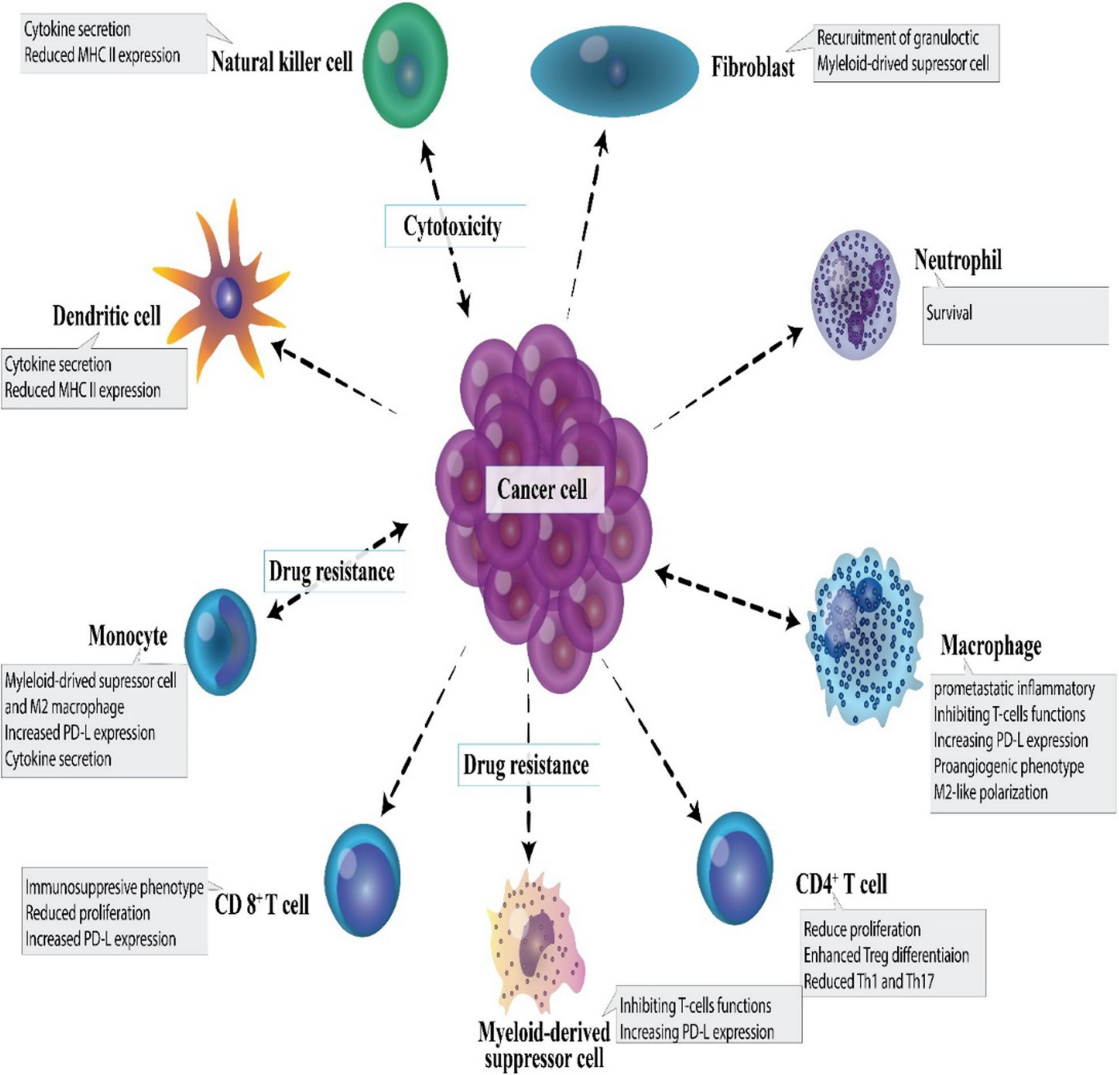
The immune polarization during cancer. The tumor milieu can participate in the immunosuppression and diminished anti-tumoral functions of different immune cells and cause the immunoinhibitory phenotype of immune cells. For example, Th cell lineages, including Th1, Th2, and Th17, during cancer have been polarized to Th2 and regulatory T cells (Tregs); consequently, CTL-mediated cancer cell cytotoxicity can be hampered via recruitment and/or conversion of Tregs and myeloid-derived suppressor cells (MDSCs) (Hu et al. 2020)

Innate immune cells such as macrophages, mast cells, dendritic cells, granulocytes, and natural killer cells are the front line of protection toward microbes and foreign causes (Table 2) (Johansson et al. 2008). In response to impaired tissue homeostasis, host tissue-resident innate immune cells regionally discharge soluble agents including chemokines, cytokines, matrix remodeling proteins, and other biologically active media that employ additional leukocytes from tissue circulation, i.e., inflammation (Johansson et al. 2008; Mirzaei et al. 2021c; 2021d). In response to a pathogen attack, specific recruited immune cells (also known as inflammatory cells) specifically kill pathogens in situ (Johansson et al. 2008). Instead, expert antigen-presenting cells (APCs) such as macrophages and dendritic cells, take foreign antigens such as tumor-associated antigens and move to lymphoid organ systems where they display their antigens to immune cells belong to the adaptive arm of the immune system (Johansson et al. 2008). Upon identification of the presented antigens, B lymphocytes, CD8+ cytotoxic T lymphocytes (CTL), and CD4+ T helper (Th) lymphocytes mount an 'acquired immune response to foreign agents (Johansson et al. 2008, 2021). These processes paved the way for stimulating antigenically engaged adaptive immune responses by severe innate immunity stimulation (Johansson et al. 2008). Once foreign agents are removed, inflammation solves, and tissue homeostasis is reconstructed (Johansson et al. 2008). Given the risk of cancer progression, it is instantly apparent that related immunological reactions needed to activate severe inflammation may be co-opted so that if prolonged inflammation is retained in the tissue, it may instead facilitate the neoplastic programming of the tissue and promote the development of tumor (Fig. 2) (Coussens and Werb 2002; Balkwill et al. 2005).

**Table 2:** Immune reactions in cancer and biofilm infection

Immune reaction	Cancer	Biofilm
Polymorphonuclear leukocytes (PMNs)	Cancer cells, via release chemokines, recruit neutrophils called tumor-associated neutrophils (TANs) with functions, including pro-and anti-cancer effects. Also, TANs can generate many agents that contribute to tumor growth, metastasis, and angiogenesis, including cathepsins, pro-angiogenic cytokines, and Matrix metalloproteinases (MMPs) (Masucci et al. 2019) Activated PMNs appear to affect cancer progression by their immunosuppressive effects and inhibition of T-cell functions (Schmielau and Finn 2001) PMN appears to be significantly involved in initiating and enhancing angiogenesis and tumor metastasis in patients with oral cancer (Jablonska et al. 2002) Unexpected antitumor effects associated with long-term employment of granulocyte colony-stimulating factor, which induces severe and persistent neutrophil stimulation, have been an easy method for solid tumors to encourage severe peritumoral PMN in tumor Sections (Souto et al. 2011) PMN-MDSCs have immunosuppressive activity and restrict immune activity in the tumor, recurrent infectious conditions, trauma, sepsis, and many pathological diseases (2018) Neutrophils, similar to macrophages, have two phenotypes, including N1 and N2, which have anti-tumor/anti-inflammatory and pro-tumor/inflammatory effects, respectively (Zhu et al. 2015; Genard et al. 2018; Fridlender et al. 2009). Although they are phenotypically different, to date, no marker is available to differentiate the phenotype of N1 from N2 in the tumor micro-environment. Reactive oxygen species (ROS) production in the N2 phenotype is very high, contributing to tumor progression in various directions (Injarabian et al. 2019)	The small contribution of PMNs to the immune response during *S. aureus* biofilm infection indicates that biofilms also circumvent significant PMN uptake by the currently unknown mechanisms (Heim et al. 2014, 2015; Hanke et al. 2013) Lysed PMN cells increase biofilm production in *P. aeruginosa* strains (Walker et al. 2005; Parks et al. 2009) When *P. aeruginosa* biofilms were formed together with PMN cells in vitro, the PMN localized to the biofilm surface but had very little microbicidal activity (Maurice et al. 2018; Rasamiravak et al. 2015) It has been found that *P. aeruginosa* utilizes PMN in a diabetic mouse model for bacterial wound infections (Watters et al. 2014)
Macrophage	One of the most abounding cells in solid tumors' environment in macrophages, these cells' presence in cancer is correlated with reduced patient survival Nielsen SR and Schmid (2017) Tumor-associated macrophage (TAMs) promotes cancer metastases via various pathways such as facilitating angiogenesis, stimulating tumor formation, and enhancing tumor cell migration and invasion (Dandekar et al. 2011) Macrophage activated by breast cancer cells has contributed to TNF-dependent stimulation of nuclear factor-B signaling pathways and c-Jun-NH2-kinase in cancer cells (Dunn et al. 2004) Macrophages exhibit several protumorigenic functions that play an essential role in cancer development and progression, such as producing cytokines and inducing tumor angiogenesis (Grivennikov et al. 2010) Macrophages enhance aggression and metastases from the primary cancer cells by their ability to join tumor cells in an autocrylic ring that promotes tumor cell migration (Wyckoff et al. 2007; Wyckoff et al. 2004)	Biofilms polarize macrophages towards anti-inflammatory phenotypes by decreasing pro-inflammatory reactions and restricting macrophages' in vivo invasion (Hanke et al. 2013; Sadowska et al. 2013) The proteinaceous factors produced by *S. aureus* (biofilm-producing) can restrain macrophage phagocytosis (2015) Macrophage dysfunction caused by *S. aureus* biofilm is partially dependent on agr (2015) Macrophages profile differential gene it has been found toward *S. aureus* biofilms (Scherr et al. 2013) Biofilm-activated M1-macrophages show that they can control biofilm infections (Yu et al. 2020)
T helper type 1 (Th1) cells	Clinical findings show that Th1/Th2 imbalances have been identified with elevated cytokines produced by Th2 in breast cancer patients (Xu 2014) Patients with a dominant Th1 response have been shown to have higher survival and lower cancer recurrence rates (Zhao et al. 2019) Changes in the response from Th1 to Th2 promote the development of breast cancer (Sherene et al. 2013) Cancer eradication is achieved by the cooperation of tumor-specific Th1 cells and tumor-penetrating antigen macrophages (Haabeth et al. 2011) Inflammation, when stimulated by tumor-specific Th1 cells, may kill cancer cells (Haabeth et al. 2011) Many immune modulators can increase the production of Th1 cytokines and boost Th1 immunity in response to cancer vaccines (Xu 2014)	An early pro-inflammatory response to Th1 and Th17, together with a down-regulated Th2 response, has been shown to arise in the initial phases of biofilm infections and may trigger tissue injury that helps *S. aureus* to be connected to and grown as a biofilm (Shirtliff et al. 2002) Biofilms indue the occurrence of CD80 and CD86, which also stimulate Th1 and Th2, respectively, suggesting the importance of a skew in the T-cell response (Slavik et al. 1999) Th1 responses may be unsuccessful in removing *S. aureus* at reduced oxygen partial pressure in the biofilm's depth (Shirtliff et al. 2002) Biofilms have been correlated with Th1 skewing in acquired immunity, while biofilm species have not been established (González et al. 2018)
T helper type 2 (Th2) cells	Th2 cells create Interleukin-4 (IL-4), and interleukin-10 (IL-10) supports tumor development by impairing the human immune response (Zhao et al. 2019) Compared to the Th1 response, the Th2 response could help cancer development (Narsale et al. 2018) The inability of Th2 cells to destroy primary cancer cells appears to be due to IL-2 deficiency, which does not allow the production of a specific anti-tumor Cytotoxic T lymphocyte (CTL) reaction (Bass et al. 1993; Erard et al. 1993) CD4 + Th2 cells mediated the tumor-suppressive effect of the thymic stromal lymphopoietin (TSLP) in these models of skin carcinogenesis (Protti 2020)	Th2 responses are effective in removing biofilm illnesses during the initial stages of biofilm growth (Shkreta et al. 2004) It was shown that in 53 Chronic rhinosinusitis patients, *S. aureus* biofilms were correlated with Th2 skewing from the acquired immune response (Foreman et al. 2011) Recent data suggest a protective role for Th2/Treg anti-inflammatory cells, as pro-inflammatory Th1/Th17 signaling, in the early development of *S. aureus* biofilm (Prabhakara et al. 2011)
CD8 T cells	Higher levels of CD8 ^+^ T cells are significantly associated with the specific survival of breast cancer (Mahmoud 2011) CD8+ T cells and CD4+ T cells were shown to have anti-tumor effects, while regulatory T cells (CD4+ CD25+ Tregs) could be accountable for the immunological hyporesponsiveness found in cancer (Sutmuller et al. 2001; Shimizu et al. 1999) In human cancer, CTL infiltration has been linked with improved clinical results and longevity in melanoma, ovarian cancer, and colon cancer Camus and Galon (2010)	Not determined
Dendritic cells	Dendritic cells (DCs) are recognized as critical players in cancer control by adaptive immunity Hansen et al. (2017) When the tumor grows early or late, depletion DCs have opposite tumor progression consequences (Scarlett et al. 2012) Only CD103^+^cDC1s were strongly associated with clinical outcomes across multiple types of cancer Hansen et al. (2017) DCs are active players that indirectly inhibit melanoma cell proliferation (Tucci et al. 2019) Under anti-tumor immune pressure, various cancer cells can develop DCs to boost immune tolerance (Wculek et al. 2020) Immune-stimulating DCs can activate strong antitumor responses during cancer immunoediting's removal and balance phases (Wu and Horuzsko 2009)	The presence of biofilms is supposed to be associated with an increase in the number of DCs responsible for presenting antigens in chronic rhinosinusitis with nasal polyposis (Karosi et al. 2013) Polymicrobial synergy in oral biofilm invades dendritic cells (El-Awady et al. 2019) Functional roles of viral biofilms include: viral transmission, escape from plasmacytoid (pDC) assays (Maali et al. 2020)
MDSC (myeloid-derived suppressor cell)	MDSCs are the most observed neutrophil-like cell community of cancer development, present in large numbers in many cancer models (Injarabian et al. 2019) MDSCs suppress antitumor immunity and promote other facets of tumor development, such as tumor angiogenesis, tumor cell attack, and the creation of pre-metastatic niches (Gao et al. 2020; Condamine et al. 2015) MDSCs are directly involved in the negative impact of patient responses to cancer therapies, including immune therapies (Diaz-Montero et al. 2009; Tada et al. 2016) The current view is that monocyte MDSCs (M-MDSCs) and PMN-MDSCs differentiate in the same monocyte and neutrophil pathways. Their spread in cancer increases with increasing GM-CSF production, CSF-1, and other growth factors (Ostrand-Rosenberg and Bronte 2012) MDSCs play an essential role in strengthening the tumor and a state of immunological anergy and tolerance Functional features MDSCs in cancer are formed by factors produced by the tumor and natural host cells (Tcyganov et al. 2018) MDSCs are significantly increased in both models of cancer mice and patients with head and neck, breast, non-small cell lungs, and kidney (Bronte et al. 2001; Nagaraj and Gabrilovich 2008; Sinha et al. 2007)	Biofilm-associated MDSCs are of a granulocyte race based on their transcriptomic profile similar to PMNs and can be classified as granulocyte MDSCs (G-MDSCs) (Heim et al. 2018) MDSCs have played an essential role in enhancing the biofilm persistence of *S. aureus*, which affects both bacterial and host-derived products (Heim et al. 2014, 2015; Scherr et al. 2014) It is not clear whether the predominance of MDSCs in biofilms is due to their active uptake by chemokines or whether these cells proliferate at the site of infection (Heim et al. 2018) In the mouse model of *S. aureus* orthopedic implant infection, MDSCs represent primary infiltration and play a key role in transforming the local environment into an anti-inflammatory environment like biofilm durability (Heim et al. 2014, 2015, 2015) The production of IL-10 by MDSCs is a mechanism used to enhance biofilms' persistence (Heim et al. 2015) Increased MDSC can be a mechanism to control the initial inflammatory response to bacteria and inadvertently pave the way to form biofilms and remain on these devices for a long time (Heim et al. 2018) *S. aureus* biofilms preferably employ MDSCs, which enhance the anti-inflammatory properties of monocytes and macrophages (2018) Decreasing MDSC improves clearance by enhancing monocyte proinflammatory activity (Heim et al. 2014, 2015)

#### Macrophage

Macrophages originate from the myeloid lineage and are part of innate immunity (Weagel et al. 2015). They are produced from blood monocytes that spread to tissues (Weagel et al. 2015). One of the key roles of macrophages is phagocytosis of bacteria and clearance of cellular debris (Weagel et al. 2015). They also contribute significantly to the induction and clearance of inflammation (Mantovani et al. 2013; Porta et al. 2009). Also, macrophages can show different reactions based on the type of stimulus they obtain from the underlying microenvironment, differing from pro-inflammatory to anti-inflammatory (Sica and Mantovani 2012). In particular, two main macrophage phenotypes have been suggested: pro-inflammatory macrophages (M1 macrophages) and anti-inflammatory macrophages (M2 macrophages) corresponding to the phenotypes of a range of responses (Weagel et al. 2015).

M1 macrophages are defensive and strongly phagocytic, contain vast quantities of reactive oxygen and nitrogen species, and facilitate Th1 immune response (Sica and Mantovani 2012). This is a macrophage phenotype typically seen throughout infectious diseases. M1 macrophages produce elevated amounts of IL-12 and IL-23, two significant inflammatory cytokines. IL-12 causes induction and clonal expansion of Th17 cells, which secrete elevated levels of IL-17 and hence lead to inflammation (Hao et al. 2012). Succinate stabilizes HIF-1α, accelerating the transition to glycolysis and increasing inflammation. Specifically, it performs this by hindering the function of prolyl hydroxylase enzymes, so preventing them from hydroxylation and destabilizing HIF-1α (Kelly and O'Neill 2015). In this case, HIF-1 may interface with coactivators to trigger the glycolytic metabolic process and to promote inflammation by boosting transcription of the IL-1β gene (Tannahill et al. 2013).

M1 macrophages are thought to perform an essential role in detecting and removing cancer cells, and their involvement typically implies a favorable prognosis (Weagel et al. 2015). Following detection of tumor cells by macrophages, various pathways can kill tumor cells, including contact-dependent phagocytosis and cytotoxicity (i.e., activation of cytokine like TNF-α) (Sinha et al. 2005). However, environmental stimuli such as tumor microenvironment or tissue-resident cells may polarize M1 to M2 macrophages (Weagel et al. 2015). The microenvironment of the tumor significantly impacts the differentiation of macrophages. The polarization mechanism can be dynamic and complex due to the great diversity in the rate of IL-10, apoptotic cells, glucocorticoid hormones, and immune complexes that may conflict with the role of innate immunity (Guiducci et al. 2005; Sica and Mantovani 2012). Notch signaling represents a vital function in the differentiation of M1 macrophages in the tumor microenvironment. It helps the transcription factor recombination signal binding protein for the immunoglobulin kappa J region (RBPJ) to control classical activation (Weagel et al. 2015). Macrophages defective in notch signaling transmit the M2 phenotype, independent of other extrinsic triggers (Wang et al. 2010). A range of stimuli give rise to the M2 phenotype, so it is essential to include the inducing molecule when contributing to these subtypes (Murray et al. 2014). The macrophages M2a are triggered by IL-13 and IL-4 (Wang et al. 2010). IL-4 and IL-13 bind to IL-4R and stimulate the JAK/STAT6 cascade that controls the expression of Chemokine (C–C motif) ligand 17 (CCL17, ARG1 (Arginase 1), IRF4 (Interferon regulatory factor 4), IL-10, Cytokine signaling suppressor 3 (SOCS3), which are genes associated with anti-inflammatory reactions (Murray et al. 2014). M2b macrophages are generally trigered by Fc + TLR/IL1-R ligands (Wang et al. 2010). Besides, these macrophages are represented by IL-10, Chemokine ligand 1 (CCL1), Interleukin-1 (IL-1), and IL-6 (Özen et al. 2011). M2c macrophages are polarized in the presence of IL-10 + TGF-β (Özen et al. 2011). They control the development of IL-10 and TGF-β and display CD163 and CD206 and many scavenger receptors (Lu et al. 2013; Chanmee et al. 2014).

#### T lymphocyte

Premalignant and cancerous tissues are reported to be correlated with improvements in the leukocyte populations and their performance blocked CTL reactions which involve in tumor rejection, in conjunction with improved humoral immunity, can facilitate tumorigenesis (Tan and Coussens 2007; Colombo and Piconese 2007). Peculiar CD4+ T-cell subsets such as Th1, Th2, and Th17 cells, release distinct collections of cytokines that mediate their reactions (Tan and Coussens 2007; Colombo and Piconese 2007). For example, Th1 cells generate, Interleukin-2 (IL-2) and Interferon-γ (IFN-γ) and direct CTL reactions. In contrast, Th2 cells provide Interleukin-4 (IL-4), Interleukin-13 (IL-13), and Interleukin-10 (IL-10) and promote local humoral immune responses, while Th17 skewed cells generate Interleukin-23 (IL-23), IL-6, and tumor necrosis factor-α (TNF-α), factors that reinforce and/or maintain persistent inflammation (Johansson et al. 2008). In peripheral blood of bladder and colon cancer cases, the balance of Th1 cells recognized by the intracellular increase of IFN-γ or IL-2 is reduced. In contrast, the proportion of Th2 cells generated by IL-4, IL-6, and/or IL-10 is considerably higher than the contrarily healthy cohorts (Agarwal et al. 2006; Kanazawa et al. 2005). Recent research examining the properties of leukocytic infiltration in human cervical cancer reported that CD3+ T-cell tumor infiltration shows heightened Th2 cytokine profiles and explicitly raised IL-4 and reduced IFN-γ (Sheu et al. 2001). In line with these results, enhancements in immune cell status (removed CTL responses and developed humoral immunity) have also been documented in persistent inflammatory disorders correlated with developed cancer risk (Tan and Coussens 2007). Overall, these persuasive clinical decisions demonstrate that improved pro-tumor immune responses underlie an elevated likelihood of neoplastic development in tissues impacted by persistent inflammatory disease pathologies and/or tissues that harbor neoplasm-initiated cells.

The precise immune reactions to the cancer are driven by encounters with mature APCs and the existence of a pro-inflammatory environment (Dunn et al. 2002). In this sense, the regulatory CD4+ T lymphocytes play a significant role in orchestrating reactions (Dunn et al. 2002). Naive T lymphocytes with the same antigen specificity can be 'polarized' into diverse functional effector cells that rely on the early environmental signal obtained when the antigen is displayed (Guy 2007). These signals are provided by innate immune cells and cells at the lesion site and are received by receptors displayed by naive T-lymphocyte precursors (Akira et al. 2006). For example, following viral infection, infected cells instantly discharge type I IFNs (IFN-α and/or β) that stimulate initial viral defense strategies and are also crucial for polarization of the immune system towards antiviral Th1 response (Levy et al. 2001; Proietti et al. 2002; Abdi and Mirzaei 2020).

Effector CD4+ T lymphocytes of Th1 progenitors have adapted to eliminate intracellular pathogens, including intracellular bacteria and viruses, by triggering CTL reactions and inducing IgG2a and IgG3 development (Weaver et al. 2006). In the sense of tumor metastasis, some IFNs have been hypothesized to mediate anti-tumor protection, in part by controlling divisive Th1 responses (Dunn et al. 2005). IFN-caused activation of Th1 cell differentiation when T-cell receptor (TCR) stimulation is followed by IFN-induced signaling via the signal transducer and STAT1 intracellular signaling cascade, the pioneer stage of Th1 differentiation (Lighvani et al. 2001). Signaling by STAT1 upregulates the formation of the Rβ2 chain of the Interleukin-12 (IL-12) receptor, thereby making cells receptive to IL-12, a cytokine essential for further differentiation of the Th1 cells (Szabo et al. 1995). This mechanism also inhibits the development of IFN-γ and provokes the expression of Interleukin-18 (IL-18) Rα receptor (Dinarello 1999). Mature Th1 effector cells may generate IFN-γ via TCR-dependent mechanisms and generate cytokines independently of antigen stimulation while being triggered by IL-12 and IL-18 (Yang et al. 1999).

Th2 lineage cells are believed to have developed to strengthen the removal of infectious diseases and are distinguished by the development of IL-4, IL-13, and Interleukin-5 (IL-5) (Lee et al. 2001). These cytokines are useful actuators of immunoglobulin E (IgE) development and recruiting of eosinophils and granulocytes (Lee et al. 2001). The configuration of Th2 cells is triggered by TCR signaling and IL-4 signaling via STAT6, occurring by epigenetic chromatin rearrangement in the Th2 cytokine bunch while concurrently restricting STAT4 and IL-12 Rβ2 expression (Reiner 2005; Ouyang et al. 2000). Together, these activities support the production of the Th2-related cytokines, making them resistant to the Th1 linear engagement's repolarization.

Recent findings have shown a greater variety in the CD4+ T-cell effector range and have related the cytokines IL-17 and IL-23 to a new branch of the Th-cell family known as Th17 cells (Mirzaei et al. 2021e; Cua et al. 2003). The production of Th17 cells from naive T cells is caused by the transformation of the growth factor-β (TGF-β) and IL-6 as an essential cofactor (Bettelli et al. 2006). TCR activation can directly induce the development of Th17 cytokines, whereas IL-1, IL-23, and IL-18 can potentiate this effect (Weaver et al. 2006). CD4+ Th17 cells have been active in removing extracellular bacteria and autoimmune diseases, such as experimental autoimmune encephalomyelitis (EAE) and collagen-induced arthritis. They are correlated with granulocyte recruitment and expression of Immunoglobulin M (IgM), IgG, and Immunoglobulin A (IgA) (Weaver et al. 2006; Annunziato et al. 2007). The Th17 responses were also related to facets of cancer growth (Langowski et al. 2006). In an in vivo model of chemically mediated skin carcinogenesis, IL-23 was considered as an essential inflammatory response mediator correlated with cancer advancement (Langowski et al. 2006).

The fourth type of CD4+ cells, called regulatory T (Treg) cells, inhibits the effector action of CTLs and performs essential physiological functions in preventing autoimmune disorder, and intensifies immunity from infections (Zou 2005; Colombo and Piconese 2007, 2020). Treg cells have been defined as normal Treg cells that develop in the thymus, and adaptive Treg cells that are distinguished in peripheral tissues (Karampoor et al. 2020). Natural Treg cells are CD4+ CD25+ Forkhead box protein P3 (FOXP3) + and shape predominantly in the thymus through high-affinity TCR with antigens expressed in the thymic stroma (Colombo and Piconese 2007; Fehérvari and Sakaguchi 2004; Weaver et al. 2006). Natural Treg cells repress immune reactions by cell covering molecules such as cytotoxic T-lymphocyte antigen 4 (CTLA4), membrane-bound TGF-β, and pericellular adenosine production (Colombo and Piconese 2007; Fehérvari and Sakaguchi 2004; Weaver et al. 2006). Adaptive Treg cells are CD4+ CD25+ FoxP3+ /low, developed in peripheral tissues in the presence of TGF-β and IL-10, which inhibit immune responses primarily by releasing soluble factors, including IL-10 and TGF-β (Weaver et al. 2006). In vivo Treg cell loss employing CD25 neutralizing antibodies augments the T-cell anti-tumor reaction and causes relapse of laboratory cancers, such as sarcomas and melanomas (Onizuka et al. 1999, 1999). Clinical reports showed that the presence of Treg cells in patients with ovarian cancer is associated with reduced survival and these results can suggest the essential function of Treg cells in controlling pro-and anti-tumor immunity (Curiel et al. 2004).

### Immune polarization in biofilm infection

**Fig. 3:**
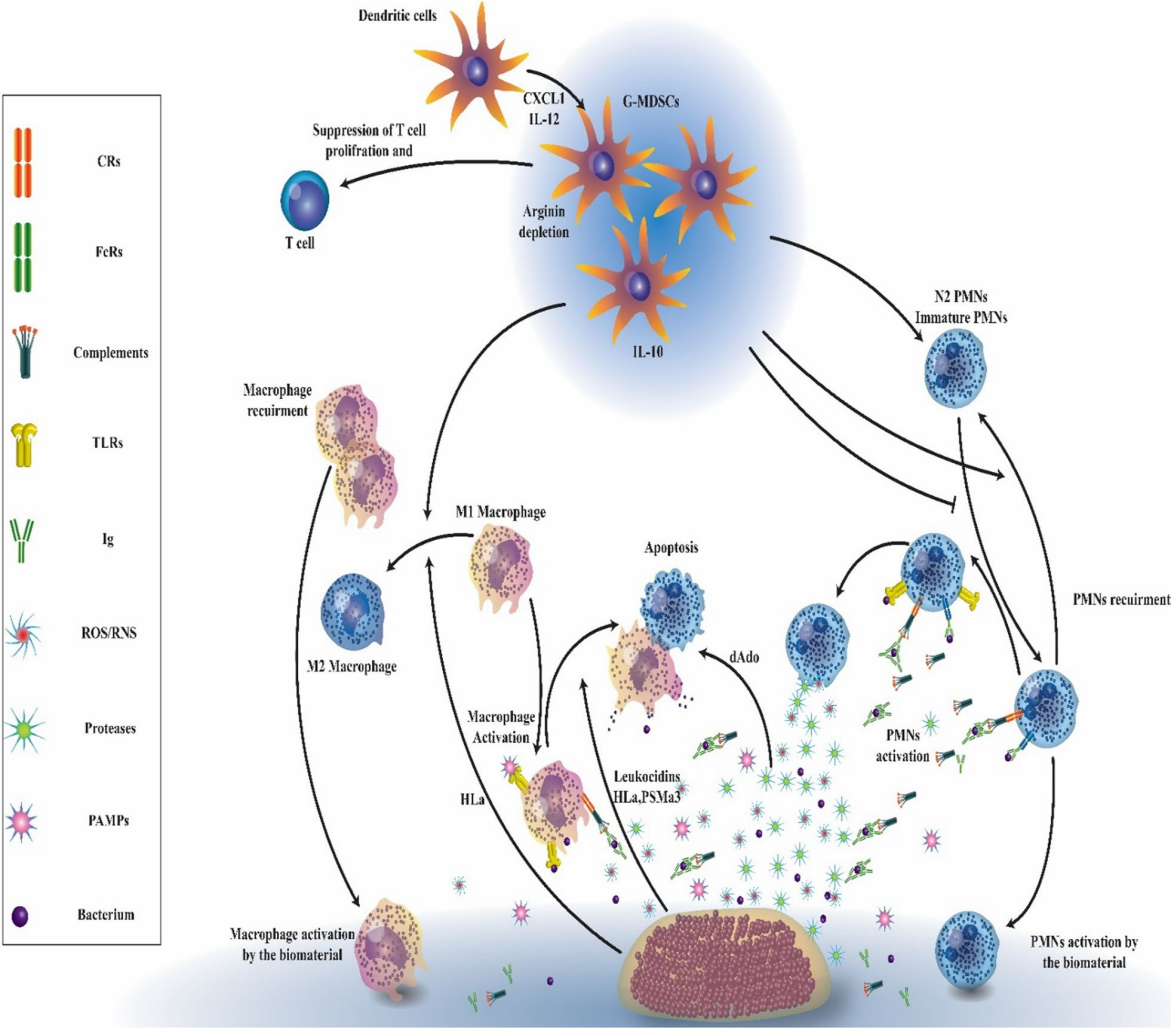
The interactions between immune cells and biofilm infection. Biofilm-associated biomaterials and bacteria interact with humoral and cellular components of the immune system. Leukocyte chemokinesis and activation are triggered by the release of pathogen-associated molecular patterns (PAMPs), opsonization of bacteria, and complement activation through the interaction with toll-like receptors (TLRs), immunoglobulin Fc receptors (FcRs), and complement receptors (CRs). Bacteria, in turn, induce the release of factors such as CXCL1, IL-12, IL-10, deoxyadenosine (dAdo), leukocidins, α-hemolysin (Hla), phenol-soluble modulin alpha 3 (PSMa3), proteases, and reactive oxygen species (ROS)/ reactive nitrogen species (RNS) that control and/or inactivate the host humoral and cellular immune responses, and also use persistence strategies, including entrenching in protective biofilms (Campoccia et al. 2019)

In chronic biofilm-associated device infections, internalized bacteria and cells in biofilms could adopt a phenotype of small colony variant (SCV), which is known for a slow rate of growth and reduced levels of secretion of toxic factors, which enable the internalized bacterial cells to survive for long periods (Bui et al. 2017; Proctor et al. 2006). The lack of diffusion of leukocytes initially described resistance against leukocytes within the biofilm and a decreased capacity of PMNs to kill the bacteria embedded in biofilm (Fig. 3) (Arciola et al. 2018). The mature biofilms have dense exo-polymeric substances that are difficult to engulf by macrophages. This leads to neutralized phagocytosis, a phenomenon that has been proposed primarily to explain the reaction of phagocytes to asbestos fibers (Thurlow et al. 2011; Donaldson et al. 2010). In vivo investigations have found that Staphylococci biofilm can deviate the innate immune responses to the pro-fibrotic and anti-inflammatory reactions, rather than the pro-inflammatory, and bactericidal reactions (Arciola et al. 2018, Hanke et al. 2012). The bacterial biofilm directs macrophage polarization from the classic pro-inflammatory phenotype to the anti-inflammatory form, the latter is characterized by the formation of anti-inflammatory mediators (Gries and Kielian 2017; Hanke and Kielian 2012). In device-associated biofilm infections, IL-12 triggers the recruitment of myeloid suppressor immune cells, which mediate the anti-inflammatory effects via their strong immunosuppressive ability and impairing phagocyte influx (Heim et al. 2015).

#### Polymorphonuclear leukocytes (PMNs)

The human immune system not only responds to the bacterial cells that contaminate medical devices, but it also responds to the biomaterial surface of the devices and senses them as the foreign body. This response stimulates inflammatory reactions that contribute to the complement system, coagulation cascade, immune cells, platelets, and particularly PMNs (Franz et al. 2011). The activation of PMNs triggers metabolic weakness and discharge of oxidative resources, resulting in the permanent release of Reactive oxygen species (ROS), which diminishes their killing ability to kill the bacterial cells (Arciola et al. 2018). The reduced bactericidal ability of PMN following exposure to the various surfaces has been demonstrated in severe biofilm-associated bacterial infections (Arciola et al. 2018). Besides the exhaustion of immune reactions due to the presence of medical devices, the bacterial cells apply various methods to evade human immunity, e.g., invasion of human cells, skewing of the immune reactions, and toxin production (Arciola et al. 2018). While many of the previous studies evaluating the bactericidal capacity of PMN were performed using planktonic cultures of bacterial cells, there has been a wave of recent works examining the interaction between bacterial biofilm and PMN. Most studies of the interactions between biofilm and PMNs were reported with the biofilms of *Staphylococcus aureus* and *Pseudomonas aeruginosa* (Alves et al. 2018).

When *P. aeruginosa* biofilms were formed together with PMN cells in vitro, the PMN localized to the biofilm surface but had very little microbicidal activity (Rasamiravaka et al. 2015a; Maurice et al. 2018). One reason for this phenomenon is that alginate polysaccharide present in the biofilm EPS matrix of *P. aeruginosa* suppresses phagocytosis and chemotaxis of PMN (Maurice et al. 2018). Rhamnolipid is another secreted component produced in *P. aeruginosa* biofilms, which antagonizes PMN cells (Jensen et al. 2010). Jensen et al. (2007) found that rhamnolipid formed by *P. aeruginosa* biofilm quickly lysed host PMNs in vitro. They showed that *P. aeruginosa* strains upregulated rhamnolipid production in reaction to the PMN exposure, indicating that *P. aeruginosa* actively senses and reacts to these immune cells (Jensen et al. 2007). Many reports show that lysed PMN cells increase biofilm production in *P. aeruginosa* strains (Parks et al. 2009; Walker et al. 2005). For example, PMN destroyed the planktonic *P. aeruginosa* strains, while biofilms increased survival (Walker et al. 2005). *P. aeruginosa* was found to use actin and DNA from the lysate of PMN to reinforce its biofilm (Gennip et al. 2012). In addition, the PMN-increased biofilm of *P. aeruginosa* was somewhat tolerant to antimicrobials. When the biofilm was exposed to DNase, it was destroyed and planktonic cells were dispersed (Walker et al. 2005). Besides, the ability of *P. aeruginosa* to benefit from PMN was recently reported in a diabetic mouse model for wound bacterial infections (Watters et al. 2014). Diabetic mice on insulin treatment were wounded and inoculated with *P. aeruginosa*, and it was demonstrated that PMNs had enhanced migration compared to the non-diabetic mouse. Nevertheless, this enhanced recruitment did not lead to enhanced clearance of bacteria. The increased migration of PMN in this mouse model resulted in the formation of *P. aeruginosa* biofilms that were enriched with DNA and showed enhanced tolerance to the aminoglycoside antibiotic gentamicin (Watters et al. 2014).

Further studies presented the disadvantageous interactions between PMNs and biofilm. In a study by Nguyen et al. (Nguyen et al. 2013), a T2 (type 2) diabetic mouse model of wound infection was used to demonstrate that the existence of *S. aureus* biofilm significantly diminished the oxidative activity of PMN, triggering a higher bacterial load and reduced rates of healing. While *S. aureus* stimulated the Neutrophil extracellular trap (NET)osis of PMN cells, it was also shown that *S. aureus* secretes enzymes that produce deoxyadenosine from NET degradation, which then stimulates the caspase 3 (Cas 3) mediated death of immune cells (Watters et al. 2015).

Additionally, clinical infections of Staphylococci are associated with diminished apoptosis of PMNs in diabetic persons, which direct the prolonged formation of TNF-α and thus reduce the bacterial clearance of PMNs (Hanses et al. 2011; Mirzaei et al. 2017; Mirzaei et al. 2020e; Rasoul et al. 2019). PMNs are impressive at killing planktonic bacterial cells; however, bacterial biofilm emerges to resist the microbicidal activities of PMNs, and can even profit from the cellular debris left behind the matrix. It is evident from these works that the interplay between biofilm and the dying and ineffective PMN contributes powerfully to the chronic inflammatory mode present in chronic bacterial infections (Watters et al. 2015). PMNs can be considered predator cells and bacterial pathogens as prey; however, the outcome does not always favor the predators, as seen with the interaction between PMNs and *Staphylococcus* biofilms. The PMN cells attack the bacteria directly and the EPS slime layer (Hänsch et al. 2012). The EPS structure and composition differ broadly among the bacterial species, and even within a single species, there is variation between strains. For example, the mucoid *P. aeruginosa* strains collected from Cystic fibrosis (CF) patients produced the alginate polysaccharide as the main EPS component, whereas non-mucoid strains did not (Jesaitis et al. 2003). Therefore, when considering the interaction between PMNs and EPS biofilm, the outcomes cannot be generalized to all pathogens. Applying the experimental findings from various animal models to human bacterial infections is relatively challenging because they do not fully represent natural human infections. For example, mice produce fewer PMNs than humans (approximately 20% of the leukocytes); hence it is feasible that the initial innate immune reactions in animals may not resemble the immune responses in humans.

#### Macrophage

Much research has focused on PMNs, but it has been shown that monocytes share many functional properties with PMNs (Hänsch et al. 2012; Jesaitis et al. 2003). Following the recruitment of PMNs to bacterial infection sites, macrophages arrive a few days later in response to the damage from infections that were not resolved by PMNs (Watters et al. 2015). The main function macrophages, namely efferocytosis, is to phagocytize the apoptotic and necrotic host cells (Khanna et al. 2010). Other macrophage functions include the formation of ROS to destroy bacteria during phagocytosis. However, the interplay of macrophages with bacterial biofilm is less well-studied than that of neutrophils. In recent years, it was shown that a quorum sensing (QS) molecule, N-acyl homoserine lactone (AHL) produced by *P. aeruginosa*, stimulated apoptosis in macrophages (Tateda et al. 2003).

Nevertheless, AHL molecules have also been demonstrated to stimulate phagocytosis in macrophages while not impacting ROS production (Vikström et al. 2005). In a work when biofilm was co-cultured with macrophages, a *P. aeruginosa* strain lacking the alginate polysaccharide was destroyed by macrophages in a few hours (Leid et al. 2005). The killing of this biofilm was attributed to phagocytosis and was dependent on IFN-γ.

Several biofilm evasion strategies have been found in the innate immunity including reductions in TNF-α, IL-1 beta (IL-1β), C–X–C Motif Chemokine Ligand 2 (CXCL2), and C–C Motif Chemokine Ligand 2 (CCL2) formation that limited macrophage invasion into the biofilm. Also, there was a skewing of the immune cells away from a bactericidal phenotype, as determined by a decrease in the formation of inducible nitric oxide synthase (iNOS) concomitant with robust ARG-1 induction (Campoccia et al. 2019, 2015; Le et al. 2018). Interactions of macrophages with biofilm show limited phagocytosis ability as seen in M2 macrophages (Campoccia et al. 2019; Thurlow et al. 2011). Although PMN is a critical anti-bacterial effector, its transcriptional ability to produce inflammatory cytokines is restricted, and because of its short life term and high cell turnover, it needs constant PMN recruitment (Campoccia et al. 2019; Yamada and Kielian 2019). By contrast, macrophages are more long-lived, produce different rates of proinflammatory molecules are important for recruitment/activation cascades of immune cells after bacterial exposure, and show potent anti-bacterial and phagocytic impacts (Campoccia et al. 2019; Yamada and Kielian 2019). Furthermore, resident macrophages are present in almost all tissues and serve as a critical primary defense mechanism against bacterial invasion by recognition via Toll-like receptors (TLRs) (Hirayama et al. 2017).

*S. aureus* biofilm triggers macrophage dysfunction in many aspects, which provide a full explanation as of why biofilm infections persist in immunocompetent individuals (Alboslemy et al. 2019; Scherr et al. 2015; Thurlow et al. 2011). However, the bacterial biofilm stimulates the influx of higher macrophages in early infection; the lack of iNOS and the massive production of ARG-1 show that bacterial cells may not be completely eradicated (Yamada and Kielian 2019; Thurlow et al. 2011). The same M2-like phenotypes were found in macrophages following biofilm co-culture (Yamada and Kielian 2019; Thurlow et al. 2011). In the second place, although biofilm was mediated to a vast accumulation of macrophages, only a few subsets migrated to the surface of the biofilm, and most produce ARG-1, which skews macrophages for ineffective killing. Latter, the data found that biofilm triggers macrophages' death by an unknown process. Notably, macrophages were capable of phagocytizing bacterial cells from the dispersed biofilm, showing that the size and inability of opsonizing intact biofilm can describe the phenotype of the dystrophic macrophages. The process that induces macrophages' death following contact with the biofilm is not well defined but can be the consequence of bacterial toxins, acidic pH, and the hypoxic microenvironment surrounding the biofilm (Lebeaux et al. 2014; Koo et al. 2017). However, findings collectively show that the biofilm re-programs macrophages responsiveness to an M2 phenotype with less anti-bacterial ability. All in all, it is proposed that biofilm attenuates the formation of inflammatory mediators and the invasion of macrophages to the site of infection. Also, the production of iNOS was mitigated, and a coincident rise in ARG-1 collectively demonstrates a skewing of the immune reactions to a pro-fibrotic M2 phenotype.

Nosocomial infection (like medical device infection) is a costly problem for the health care system, and strains of *S. aureus* (in particular methicillin-resistant *S. aureus*) are the predominant species that cause this infection (Haque et al. 2018, 2012). The medical device infection is associated with the ability of *S. aureus* to attach to the host coating devices by host protein via cell wall bacterial adhesins. Biofilm of *S. aureus* makes eradication of the infection difficult because of increased resistance of the embedded bacterial cell to antibiotics and immune reactions (Hanke et al. 2012). Some studies have found an effect on IL-1β in biofilm infection, whereas TLRs had no influence (Hanke et al. 2012). Interestingly, both IL-1 receptors (IL-1R) and TLRs are involved in signaling pathways related to Myeloid differentiation primary response 88 (MyD88). However, the role of this crucial factor in modulating response to biofilm infection is unclear (Hanke et al. 2012). A study by Mark et al. (2012) evaluated the biofilm infection by *S. aureus* in MyD88 knockout mice. In the study, MyD88 knockout mice showed more bacterial infection compared to wild-type mice. Production of inflammatory mediators such as IFN-c, IL-6, and Chemokine C–X–C motif ligand 1 (CXCL1) was significantly reduced in MyD88 knockout animals. Tissue staining for biofilm by the immunofluorescence technique showed an increased fibrosis in these mice, coinciding with increased recruitment of M2 macrophages. According to previous works on TLR2, TLR9, and IL-1β knockout mice, the report of Market al. (2012) suggests that MyD88 signaling is a practical pathway in regulating fibrosis and the polarization of macrophages in biofilm formation. In sum, the current data indicate that the absence of MyD88 (not “signaling mediated to MyD88”) exacerbates the polarization of M2 and fibrosis in the biofilm infection of *S. aureus*, resulting in the growth and propagation of bacteria during acute infection. Taken in the context of previous studies with TLR2 and TLR9 knockout animals, where these indices were not affected, the recent studies can enhance our understanding of MyD88-dependent cascades, indicating the production of inflammatory mediators that do not involve TLRs (Hanke et al. 2012). Therefore, we note that new approaches to disrupt the signaling process mediated by MyD88 could be effective in the treatment of biofilm infection.

### MDSC

MDSC is a subset of immature granulocytes and monocytes that are intermediates of common myeloid differentiation and development (Heim et al. 2015; Gabrilovich and Nagaraj 2009). In a normal situation, MDSCs differentiate at the location of inflammation to form mature myeloid populations, including dendritic cells, PMN, as well as macrophages (Gabrilovich and Nagaraj 2009; Dai et al. 2015). However, under pathological conditions, like chronic inflammation, tumors, and bacterial biofilm-associated infections, MDSC is arrested in an immature mode, regulating inflammatory mechanisms by its repressive actions (Youn et al. 2012; Ostrand-Rosenberg and Sinha 2009). MDSC proliferation in cancer is caused by various growth factors and cytokines, like Granulocyte–macrophage colony-stimulating factor (GM-CSF), IL-6, vascular endothelial growth factor (VEGF), and Granulocyte-colony stimulating factor (G-CSF) (Ostrand-Rosenberg and Sinha 2009). Nevertheless, it is unclear whether the host or bacterial components release MDSC in the biofilm infection or arrest it in its immature state.

Following the development of MDSCs, inflammatory stimuli generate and cause immunosuppressive effects (Condamine and Gabrilovich 2011). IL-12 has been shown to facilitate MDSC recruitment in the *S. aureus* biofilm, which may be an indirect impact, as this cytokine is not a chemo-attractant agent (Heim et al. 2015). Nevertheless, IL-12 cytokine is not required for the activation of MDSCs in the biofilm infection since MDSCs from both p35 knockout, and IL-12 p40 mice still inhibit the proliferation of CD4+ T cells (Heim et al. 2015). Hence, other inflammatory agents should contribute to the production of IL-10, ARG-1, and other anti-inflammatory components formed by MDSCs with immunosuppressive activities against the *S. aureus* biofilm (Heim et al. 2015). IL-10 is a well-defined anti-inflammatory cytokine that controls inflammatory reactions, e.g., suppressing the activation and polarization of T cells and production of IL-10 by MDSCs promotes the polarization macrophages to the anti-inflammatory state (Bunt et al. 2009; Murray 2005, 1993). MDSCs are crucial in the anti-inflammatory reactions against *S. aureus* strains and enhance the chronic infection (Heim et al. 2014). Biofilm-associated infection is known to hijack the innate immune response towards an anti-inflammatory mode (Fig. 3) (Heim et al. 2014). In this regard, IL-10 can facilitate the development of persistent infections and subvert mechanisms of immune eradication (Heim et al. 2014). Although the production of IL-10 by MDSCs has been implicated as an immunosuppressive mechanism, so far, there is no report demonstrating that the activity of MDSCs is mediated to IL-10 in biofilm infection. A study by Heim et al. (2014) found that MDSCs produce massive levels of IL-10 against the biofilm mode of growth, which restricts proinflammatory gene expression of monocyte and is directly involved in the biofilm persistence of *S. aureus* in later phases of infection. Although there are few reports of T-reg cells involved in the *S. aureus* biofilm, the production of IL-10 by MDSCs caused stimulation of T-reg cells that could generate IL-10, which may perpetuate the anti-inflammatory state (Heim et al. 2014). In future works, the mechanism of accumulation, proliferation, and activity of MDSCs in the biofilm infection must be examined.

Depending on the role of IL-10 in biofilm infections, *S. aureus* components that increased IL-10 synthesis by MDSCs and macrophages were discovered, such as genes that are involved in lactate synthesis, implying that bacterial lactate is an important determining factor of leukocyte activation (Heim et al. 2020). Lactate from *S. *aureus biofilms serves as a metabolic virulence factor, increasing IL-10 release in MDSCs and macrophages via blocking Histone deacetylase (HDAC). This reduces leukocyte pro-inflammatory function, which is one mechanism that accounts for biofilm persistence. This is similar to tumor cells generating lactate in the tumor microenvironment, promoting the development of MDSCs and immunosuppression. A study conducted by Husain et al. (Husain et al. 2013) discovered that increased lactate synthesis by tumor cells due to high glycolytic metabolism inhibited host immunological response to tumor cells through modulation of MDSCs function.

## Immunometabolism in cancer and biofilm infection

### Cancer

**Fig. 4:**
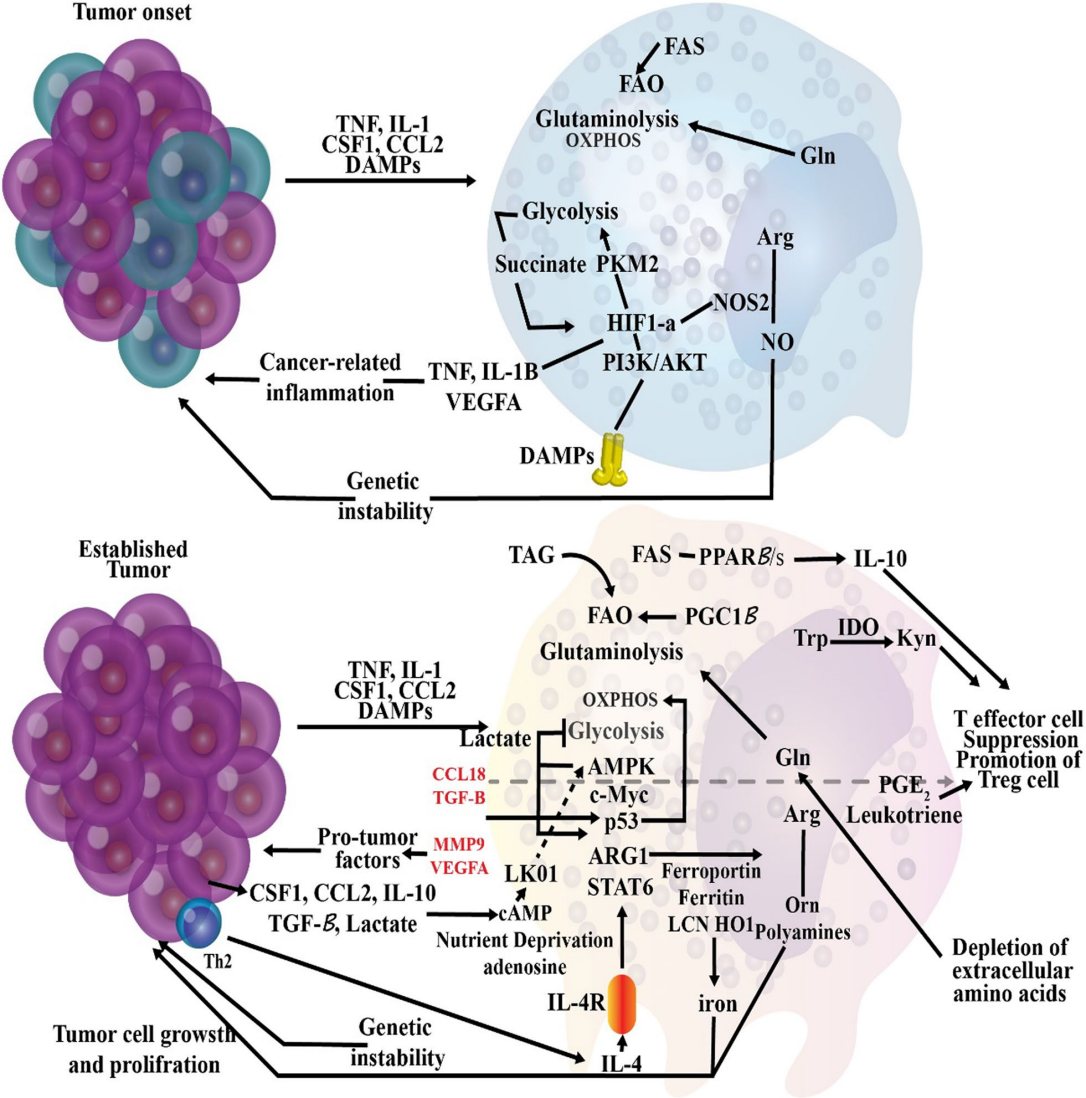
Metabolic reprogramming of immune cells and their altered functions to cancer cells. A) Metabolic shift in tumor-associated macrophages (TAMs) at tumor onset and during progression. At tumor onset, a glycolytic shift, activation of HIF1α, and inhibition of oxidative phosphorylation (OXPHOS) induce the expression of NO, ROI, and inflammatory cytokines such as IL-1β and TNF in inflammatory macrophages to support cancer-related inflammation and genetic instability that leads to tumorigenesis. At established tumors, nutrient deprivation-induced AMPK activation, Th2-derived IL-4 (which activates STAT6, p53, c-Myc, and PGC1β), lactate accumulation, and PKM2 activation suppress glycolysis in TAMs while upregulating OXPHOS. This metabolic change induces immunosuppressive TAMs that promote tumor growth. Changes in the metabolism of iron, amino acid, and fat that contribute to this process are also shown

**Fig. 5:**
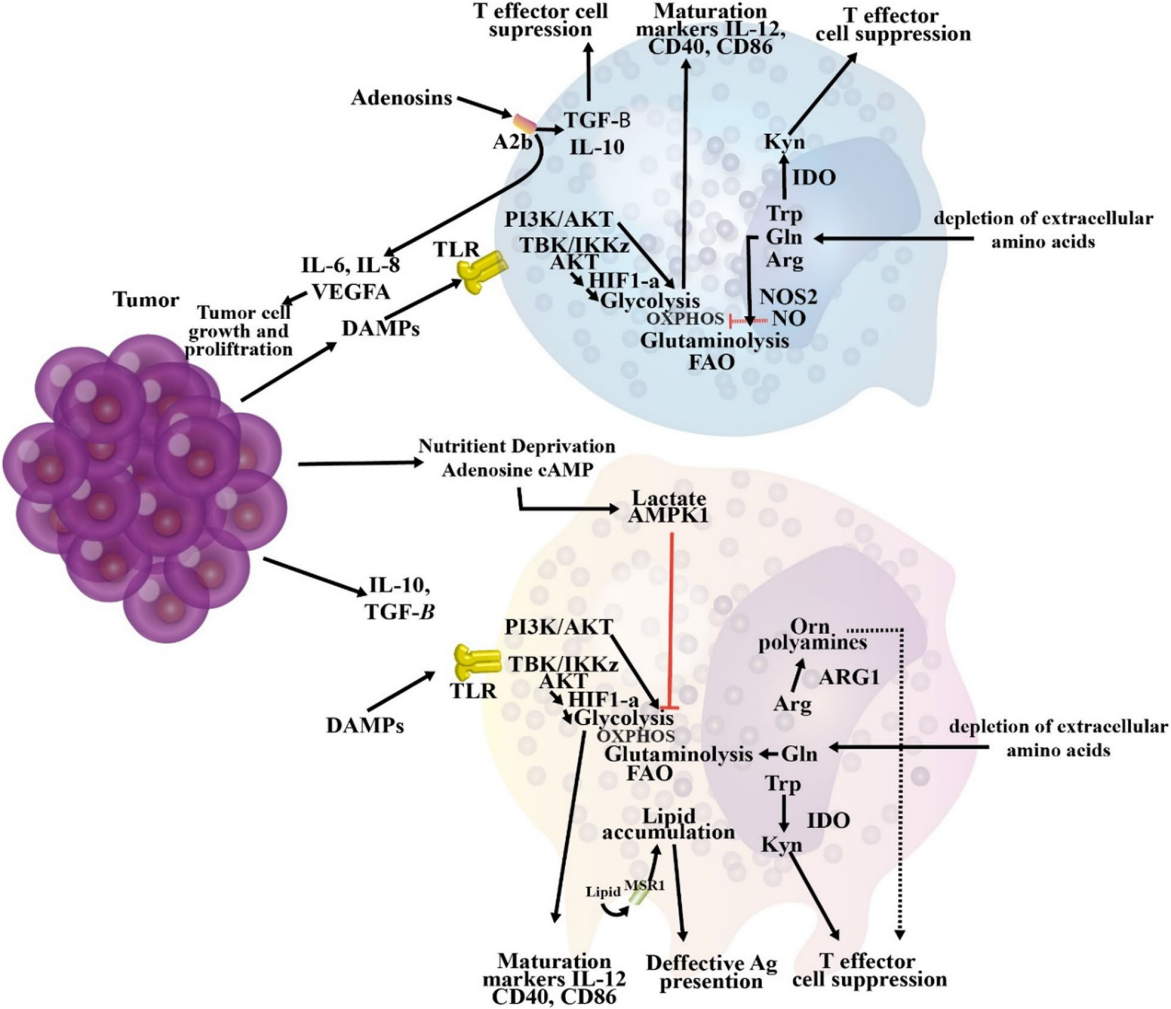
Metabolic alterations in tumor-associated dendritic cells (TADCs) during tumor progression. TADCs encounter hypoxia and tumor-derived damage-associated molecular patterns (DAMPs) to upregulate glycolysis via an initial TBK1-IKKε pathway and/or later PI3K-AKT-HIF1α pathway. HIF1α impairs the maturation of DC cells and upregulates the expression of A2b and NO (which inhibits OXPHOS). A2b-adenosine interaction induces immunosuppressive cytokines (e.g., TGF-b and IL-10) and pro-tumor cytokines (e.g., IL-6 and IL-8) that promote tumor growth. Nutrient deprivation-induced AMPK activation and lactate accumulation suppress glycolysis and upregulate OXPHOSP in TADCs. In addition, amino acid uptake, metabolism, and lipid accumulation promote immunosuppressive events that lead to tumor growth

**Fig. 6:**
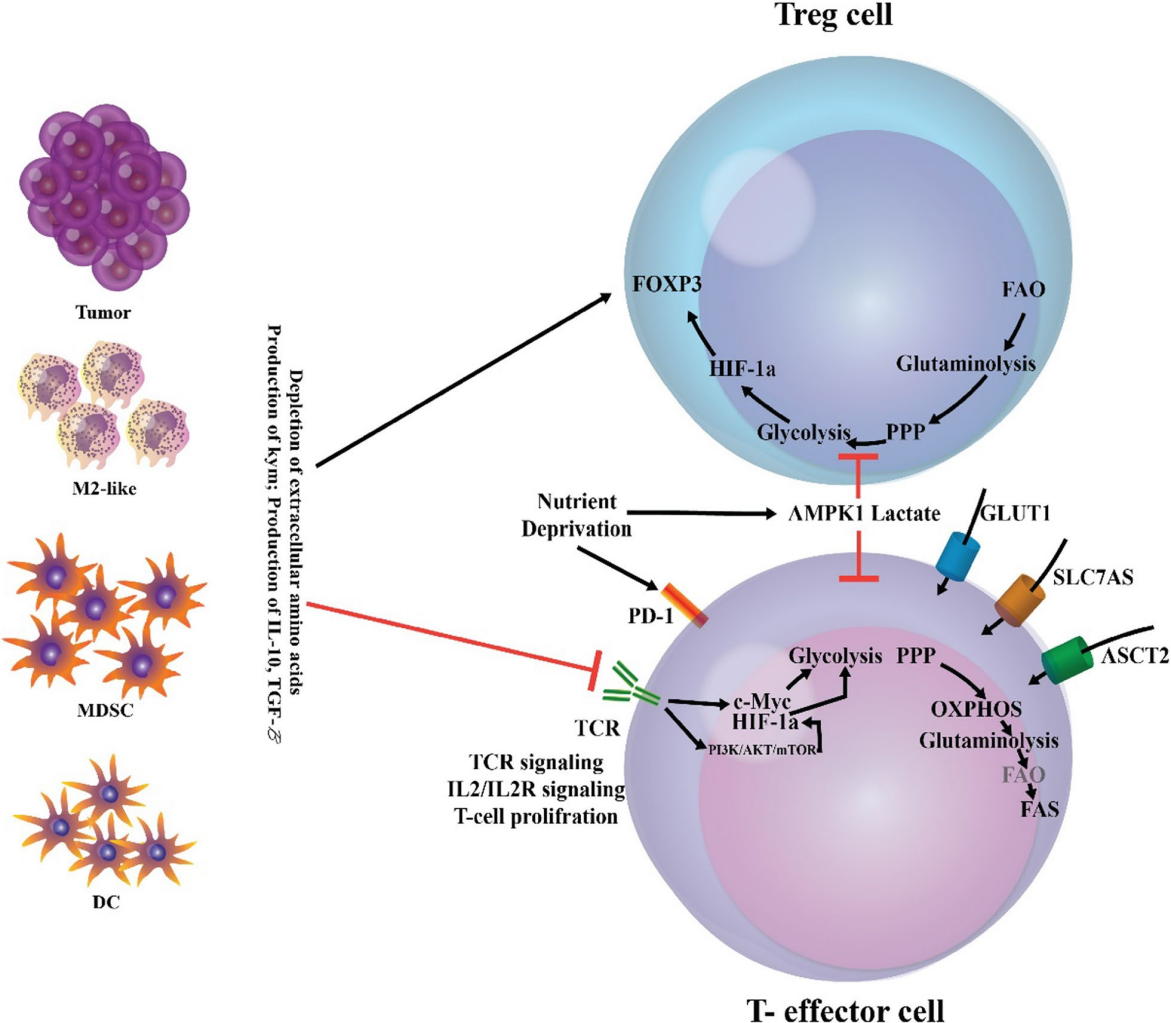
Inhibition of T effector cells and activation of Treg cells by the tumor microenvironment. Nutrient deprivation-induced AMPK activation, depletion of extracellular amino acids, and lactate accumulation inhibit TCR signaling and downstream glycolysis that inhibits T effector cells. Inhibition of glycolysis, activation of OXPHOS, fatty-acid oxidation (FAO), and tumor microenvironment-related factors (e.g., hypoxia, Kynurenine (Kyn), TGF-b, and IL-10) instead activate Treg cells, which causes immune evasion and tumor growth (Biswas 2015). Arg, Arginine; Orn, Ornithine; Trp, tryptophan; Gln, glutamine; NO, nitric oxide; FAS, Fatty acid synthase

Metabolic reprogramming of Tumor-associated macrophages (TAMs), DCs, and T cells and their altered functions during tumor progression are dipticated in Figs. 4, 5, 6. In this section, we overview the immuneometabolism reactions in some immune cells during cancer (Table 3).

**Table 3:** Metabolic changes in immune cells during cancer and biofilm infection

Cell	Metabolic reactions in tumor	Metabolic reactions in biofilm
Neutrophils	Neutrophil metabolism is poorly defined in the sense of cancer, and the metabolism of neutrophil-specific subgroups (i.e., N1 or N2) is understudied. In the sense of inflammation, neutrophils need an improved flow of glycolysis and pentose phosphate metabolism (PPP) to support nucleotide synthesis, respiratory bursting, and Neutrophil extracellular trap (NET) development, with minimal criteria for the tricarboxylic acid (TCA) cycle, oxidative phosphorylation (OXPHOS), and fatty acid synthesis (FAS) Glucose absorption, glycolysis, and metabolic change to PPP are significant for neutrophil netting sequesters circulating tumor cells and promoting metastases. The data provide some suggestions about how the metabolic shift in neutrophils by controlling functions such as NETs can lead to cancer development (Biswas 2015) Together, tumor development and the acquisition of immunosuppressive neutrophils are concurrent with a complex development of their metabolism and nutrient utilization, spanning from glucose-fueled glycolysis and OXPHOS to fatty acid-fueled Fatty acid oxidation (FAO) in the initial and end stages, respectively (Riera-Domingo et al. 2020)	It has been found this diminished hypoxic levels of oxygen result in diminishing neutrophil killing ability and because this ability is mediated by the formation of reactive oxygen species (ROS) from oxygen (Jesaitis et al. 2003, 2018; Mandell 1974). Also, suppose this hypoxic situation is continued. In that case, neutrophils will be impaired in producing ROS like hypochlorous acid and hydrogen peroxide, causing impairment of the neutrophil's ability to eliminate and control biofilm infection (Wu et al. 2018) PMNs in the cystic fibrosis (CF) airway of patients has increased the generation of specific metabolic and stress pathways such as CD39, CD114, C–X–C motif chemokine receptor 1 (CXCR4), as well as Receptor for Advanced Glycation Endproducts (RAGE) (Makam et al. 2009). In this regard, it has been noted that a shift in the formation of their nutrient transporters influences the inorganic phosphate and glucose transports that are part of their adaptation to the airway of CF patients (2013) Of note, about of anaerobic situation in the mucus of CF patients, Kolpen et al. (Kolpen et al. 2010) hypothesized that the respiratory burst of the polymorphonuclear leukocytes (PMNs) involves oxygen consumption. The interactions between PMNs and microorganisms can enhance oxygen consumption because of extra respiration during phagocytosis by the respiratory burst resulting from the electron reduction of oxygen by NADPH oxidase to O-2 (Baldridge and Gerard 1933; Babior et al. 1976)
Macrophage	Throughout tumor initiation, inflammatory macrophages by glycolytic change, HIF1a (Hypoxia Inducible Factor 1 Subunit Alpha) induction, and blocked OXPHOSP mediate NO (nitric oxide), ROI, IL-1b, and TNF expressions to promote genetic instability cancer-related inflammation that contributes to tumorigenesis (Biswas 2015) The HIF1a-induced expression of the VEGFA angiogenic molecule is also seen. In TAMs of advanced tumors, activation of AMP-activated protein kinase (AMPK) through nutrient deprivation, lactate accumulation, and Th2-derived IL-4 (activates c-Myc, p53, STAT6, PGC1b), and activated PKM2 suppresses glycolysis while up-regulating OXPHOSP. This induces immunosuppressive macrophages that stimulate tumor development. Changes in the metabolism of amino acids, iron, and facts that lead to this mechanism are often suggested (Biswas 2015)	Ammons et al. (2017), via metabolomics by 1D 1H Nuclear Magnetic Resonance (NMR) along with immunologic evaluations, showed substantial overlap between the metabolic profile driving macrophage polarization either into the M1 phenotype or the M2 phenotype and *P. aeruginosa* biofilm included crucial metabolic pathways that modulate immunomodulation in macrophages like shifts between the PPP and TCA cycle and amino acid metabolism. They found that exposure of non‐polarized macrophages to small metabolites from planktonic- and biofilm mode of *P. aeruginosa* resulted in distinct metabolic pathways involved in macrophage immunomodulation include increased glycolysis, uncoupling of the TCA cycle, and selective uptake and metabolism of amino acids Prendergast et al. (2017). Taken together, they found that *P. aeruginosa* biofilms relevant to specific pathological influences toward macrophages via coordinated metabolic interplays, which cause immune polarization and can involve deviation from the usual healing process of wound and expansion of a chronic host wound Prendergast et al. (2017) The inflammatory status of leukocytes is associated with their metabolic situation. In this regard, in a study by Yamada et al. (Yamada et al. 2020), monocytes associated with biofilm show a metabolic favoring to OxPhos and less aerobic glycolysis for prompting their anti-inflammatory role that causes persistence of *S. aureus* biofilm Fuchs et al. (2020) found some metabolic pathways that are differentially increased in planktonic- and biofilm-exposed macrophages, such as purine biosynthesis glycolysis, branched-chain amino acid catabolism, inositol phosphate metabolism, as well as glycerol metabolism (Fuchs et al. 2020). Besides, these metabolic patterns show that biofilm-exposed macrophages have a hyper-inflammatory metabolic profile, diminished glycerol metabolism, and increased catabolism of amino acids and lactate compared to the planktonic exposed macrophages (Fuchs et al. 2020)
T cells	High expression of the glycolysis pathway in tumor cells reduces amino acids and nutrients and disrupts T cells' antitumor function (Biswas 2015). Under these conditions, as Treg cells rely more on the FAO than glycolysis, they expand and modulate their immune system (Macintyre et al. 2014; Noman et al. 2014). Also, lactate produced in tumor cells similar to the kynurenine (an amino acid metabolic product) suppresses T cells' antitumor activity and at the same time increases the Treg cells' (Siska and Rathmell 2015). Besides, the HIF1a produced by tumor cells increases Treg cell expansion and the induction of programmed death-1-ligand 1 (PD-L1) (Noman et al. 2014; Ben-Shoshan et al. 2008) The decline of extracellular amino acids (through tumor absorption and other cell types), lactate aggregation, and nutritional starvation AMPK activation prevents T-cell receptor (TCR) signaling and downstream glycolysis (indicated by red T-symbol) in T-cells (e.g., CD8 ]+ cytotoxic T-cells). This resulted in the repression of the propagation and action of the effector. Inhibitory effect of AMPK glycolysis, stimulation of OXPHOS and FAO, and microenvironment-derived tumor stimuli (e.g., Transforming growth factor-beta (TGF-β), Interleukin 10 (IL-10), Kyn, and hypoxia) instead facilitate the growth of regulatory T cells (Tregs). This encourages immune evasion and tumor development (Biswas 2015)	Not determined
Dendritic cell	Tumor-associated dendritic cells (TADCs) encounter tumor-derived Damage-associated molecular patterns (DAMPs) and hypoxia to upregulate glycolysis through initial TBK1-IKKε and/or later PI3K-AKT-HIF1a pathway. HIF1a impairs DC maturation and raises A2b and NO production (which inhibits OXPHOSP). Adenosine-A2b interaction causes immunosuppressive and pro-tumor cytokines. Rising tumor gradually results in nutrient deficiency that triggers AMPK in TADCs. This, along with lactate aggregation, contributes to potential glycolysis inhibition and improved control of OXPHOSP in TADCs. The absorption of amino acids from the tumor microenvironment, metabolism, and MSR1-mediated lipid aggregation further facilitates immunosuppressive activities to help tumor development (Biswas 2015)	Not determined
MDSCs	Although the critical role of nitrogen metabolism in the control of immunomodulatory activities of MDSCs in tumor situations is well-founded, very little is understood about the other metabolic processes in such cells. Enhanced carbon metabolism (glycolysis, glutaminolysis, and TCA activity) and its crosstalk with arginine metabolism were observed during MDSC maturation. However, its association with both AMPK and Sirtuin (SIRT) enhanced expression, which is considered to interact with glycolysis, requires clarity. Increased fatty acid absorption and the FAO have also recently been shown to control tumor infiltration MDSC's (Hammami et al. 2012; Liu et al. 2014; Hossain et al. 2015)	Not determined

#### T cells

Immune cells such as stimulated cytotoxic T cells experience complete metabolic changes to conduct effector roles, such as destroying tumor cells and secreting cytokines (Table 2) (Singer et al. 2018). Aerobic glycolysis, a mechanism in which glucose is metabolized to pyruvate and eventually to lactate in a sequence of enzymatic reactions that generate ATP and substrates for other metabolic processes, was first described in cancerous cells (Singer et al. 2018). Intriguingly, non-malignant proliferating cells undergo aerobic glycolysis, which is also deemed critical for optimum T-cell activity (Cham et al. 2008). Nevertheless, T cells are metabolically versatile, and aerobic glycolysis can not be essential to their stimulation and preservation (Singer et al. 2018; Cham et al. 2008). On the other hand, Glycolysis is vital for T-cell replication (Renner et al. 2015).

Under normoglycemic circumstances, T cells upregulate the main glucose converter Glucose transport protein type 1 (GLUT1), accompanied by enhanced glucose absorption and glycolysis following antigenic stimulation (Frauwirth et al. 2002; Macintyre et al. 2014). Also, pyruvate, one of the final metabolites of glycolysis, is primarily reduced to lactate rather than oxidized in mitochondrial respiration (Singer et al. 2018). However, T cells might not even be able to preserve a persistent status of the glycolytic pathway that is essential for their effector role (Singer et al. 2018). Aerobic glycolytic pathway contracts and memory T cells, which are less reliant on glycolysis and more participate in mitochondrial respiration, occur throughout acute infections (Buck et al. 2015; MacIver et al. 2013). On the other side, cancer cells can sustain and ultimately improve elevated glucose absorption and glycolysis, reducing intratumoral glucose levels (Voelxen et al. 2018; Battista et al. 2016).

In contrast, glucose deficiency will directly inhibit the production of IFN-γ, a crucial T-cell effector molecule in CD8+ T cell tumor infiltration (Chang et al. 2015). It has been suggested that these results be facilitated by glyceraldehyde-3-phosphate dehydrogenase (GAPDH), a crucial anaerobic glycolysis enzyme that may also influence post-transcriptional alteration of mRNAs (Singer et al. 2018). When T lymphocytes perform high glycolytic pathway speeds, GAPDH is dedicated to their metabolic function (Singer et al. 2018). However, in the sense of lower glycolytic flux, GAPDH inhibits the translation of IFN-γ (Chang et al. 2013). It has also been reported that glucose deficiency inhibits TCR-dependent Ca2+ Nuclear factor of activated T cells (NFAT) signaling, contributing to T-cell hypo-responsiveness (Singer et al. 2018). CD4+ Treg cells can suppress inflammation, often correlate with cancer development, and impede local anti-tumor defense (Singer et al. 2018). Of note, as demonstrated in murine models, Treg cells produce low rates of GLUT1, are not depend on glucose absorption and glycogenesis, and, like to CD8+ memory T cells, conduct OxPhos and lipid oxidation (Michalek et al. 2011). FOXP3, the lineage-defining transcription factor for murine Tregs, was suggested as the primary regulator of this phenotype (Gerriets et al. 2016). Mechanically, FOXP3 stimulates the development of genes which are required for synthesis synthesize of lipids and peptide hormones (Singer et al. 2018). It also decreases the control of genes involved in glucose absorption and glycolysis (Singer et al. 2018). Notably, the forced expression of FOXP3 prevents the phosphatidylinositol 3-kinase (PI3K)/protein kinase B (AKT) signaling cascade of Mammalian target of rapamycin complex 1 (mTORC1) implicated in the activation of glycolytic machines (Gerriets et al. 2016). Contradictory, glucose excess may be necessary for Treg initiation, as glycolysis in traditional CD4+ T cells is required for the induction of a regulatory phenotype in Treg cells by translocation of the glycolytic enzyme enolase-1 (ENO1) to the nucleus where it attaches to FOXP3 regulatory regions, such as its promoter and its Conserved non-coding sequence 2 (CNS2) (Rosa et al. 2015). These findings demonstrate that glucose is essential for the growth of Treg cells, for example, in the lymphatic system. Following the invasion of cancer, glucose may support Treg preservation and performance.

#### Neutrophils

Neutrophils rely on the glycolysis pathway and the PPP to perform their function; the PPP produces NADPH, a cofactor of NADPH oxidase, a key enzyme associated with the anti-microbial activity of neutrophils (Kumar and Dikshit 2019). Assessments of glucose-6-phosphate transporter dysfunction in neutrophils have demonstrated that glycolysis has a role in controlling important neutrophil functions, such as oxidative bursting and chemotaxis (Kumar and Dikshit 2019). An essential feature of neutrophils is the development of NETs, a combination of DNA, histones, and antimicrobial peptides that traps and destroy microbes (Kumar and Dikshit 2019). Glucose absorption, glycolysis, and metabolic changes in the PPP are essential factors for developing NETs (Kumar and Dikshit 2019; Azevedo et al. 2015). NETs can capture circulating tumor cells and facilitate metastases (Kumar and Dikshit 2019; Azevedo et al. 2015). NETs have also been found to accumulate in tumor-bearing mice vascular systems correlated with pro-inflammatory adhesion molecules and cytokines that lead to tumor-induced organ dysfunction (Kumar and Dikshit 2019; Azevedo et al. 2015). All in all, these data include some insights about how the metabolic shift in neutrophils by controlling functions such as NETs could lead to cancer progression. Further characterization of cancer-related neutrophil activities and its control by intrinsic cell metabolism is now needed.

#### Macrophages

TAMs have been shown to accumulate in the existence of hypoxia-inducible factor 1 alpha (HIF1α) in hypoxia environments (Henze and Mazzone 2016; Biswas 2015). Since specific glycolytic genes, such as GLUT1, Hexokinase 2 (HK2), 6-phosphofructo-2-kinase/fructose-2,6-bisphosphatase-3 (PFKFB3), and Phosphoglycerate kinase 1 (PGK1), are overexpressed, TAMs tend to use the glycolysis in environments undergoing hypoxic conditions, contributing to the development of inflammatory phenotypes that promote tumorigenesis (Biswas 2015). Latest metabolic screening has shown a preferred accumulation of many metabolites and overexpression of genes of glutamine synthesis (glutaminolysis) pathways (e.g., glutamate, alpha-ketoglutarate (AKG), Glutamic pyruvate transaminase (GPT2), and Glycine amidinotransferase (GATM)) in IL-4-induced M2 macrophages (Biswas 2015). In such cells, glutamine was necessary to protect the productive TCA cycle and the Uridine diphosphate N-acetylglucosamine (UDP-GlcNAc) formation process needed for N-glycosylation. Consequently, glutamine controlled the expression of M2 macrophages markers such as the CD206 and IRF4 N-glycosylated receptor, Kruppel Like Factor 4 (KLF4), and C–C Motif Chemokine Ligand 22 (CCL22). Corroborating these decisions, TAMs from Lewis lung carcinoma, which exhibit M2 phenotype, also revealed elevated gene expression of glutamine pyruvate transaminase (GPT) and glutamate-ammonia ligase (GLUL) (Biswas 2015). In the environment of cancer, macrophages adjust their lipid profile. The Lewis lung carcinoma research revealed distinct lipid profiles in macrophages and cancerous cells (Chen et al. 2021). Leukotrienes (Leukotriene B4 (LTB4), Leukotriene C4 (LTC4), and Leukotriene D4 (LTD4)) were derived primarily from myelomonocytic cells, while prostaglandins (Prostaglandin E2 (PGE2), Prostaglandin D2 (PGD2), Prostaglandin F2 (PGF2)) were produced from both myelomonocytic and cancerous cells. In comparison, alveolar macrophages and infiltrating TAMs varied in eicosanoid profiles, the previous expressing Cyclooxygenase-1 (COX1) and 5-lipoxygenase (5-LOX) leukotrienes, whereas the latter expressed COX2 and prostaglandins.

#### B cells

Compared with T lymphocytes, B lymphocytes are highly metabolically active (Singer et al. 2018, Goodarzi et al. 2020). Each phase has a unique reliance on glucose metabolism during B cells' growth, and pre-B cells are much less glucose-dependent than immature B lymphocytes (Kojima et al. 2010). After stimulation, naive B lymphocytes propagate and enhance glucose absorption and lactate output, close to what happens with T-cell stimulation (Garcia-Manteiga et al. 2011). In line with this, substantial B-cell metabolic reprogramming is needed for development of antibodies (Caro-Maldonado et al. 2014). Recently, Jellusova et al. (Jellusova et al. 2017) identified the need for high glycolytic action of germinal center B cells to sustain their development and spread in the hypoxic microenvironment. In addition to glycolysis, an improvement in mitochondrial content has been reported in germ-center B cells (Jellusova et al. 2017). In conjunction with hypoxia in the cancer microenvironment, glucose deficiency can likely benefit those B-cell phenotypes, producing an immunosuppressive environment.

### Biofilm

The host immune reactions are very dynamic in function and growth because they react to immune problems and adopt the distinct metabolic settings that allow them to balance their need for energy, longevity, and molecular biosynthesis (Loftus and Finlay 2016). During immune reactions, immune cells could pass through several tissues containing various nutrients and oxygen. These cells modify their functional activities dramatically in response to activation; for example, lymphocyte cells transform to produce an inactive mode into a high proliferation and growth cell. In some situations, they form a high rate of cytokines (Loftus and Finlay 2016). These functional and micro-environmental changes in immune cells indicate significant metabolic stress but are dynamically managed by reprogramming the metabolism. Most tissues are vascularized and, as a result, filled with oxygen micro-nutrients (Loftus and Finlay 2016). In immune reactions, the local environment can change because of competition for oxygen and micronutrients. As well-seen in tumor cells, they have a tremendous appetite for micronutrients and glucose; hence, they reduce the rate of glycolysis in infiltrating lymphocyte cells in the tumor (Loftus and Finlay 2016). On the other hand, microbial infections can compete with the host immune cells for oxygen, glucose, and micronutrients (Loftus and Finlay 2016). *S. aureus* infections can lead to localized hypoxia because of increased oxygen consumption by bacterial and immune cells (Vitko et al. 2015). Additionally, viruses can rearrange infected host cells to regulate glucose uptake and metabolism to facilitate virus replication; that way, the level of glucose can decrease in immune cells during viral infections (Thai et al. 2014; Ripoli et al. 2010). In addition, different cells in inflammation sites can liberate enzymes including ARG and indolamine-2, 3-dioxygenase that consume micronutrients in the peripheral niches (Hirayama et al. 2009).

One of the most usual conditions of biofilm in oxic situations is a gradient of oxygen concentration, that is, at the surface layers of the biofilm, respiring bacterial cells use oxygen resulting in an oxygen limitation condition that changes the bacterial metabolism and provides anaerobic situations inside lower layers of EPS (Stewart 2003; Wu et al. 2018). Additionally, a growing body of documents shows oxygen limitation and anaerobic metabolism changes (Wu et al. 2018). For example, hypoxia situations were observed in non-healing wounds infected with obligate anaerobic microorganisms such as Clostridia and Bacteroides, which are usually isolated in chronic wounds (Dowd et al. 2008; Frank et al. 2009). In this regard, these bacteria, which require highly reduced conditions to be able to multiply, produce anoxic niches in these infected sites. Besides, direct measurement of oxygenation of skin tissue in the proximity of chronic wounds has been found and proposed to mediate the host (Ruangsetakit et al. 2010). Additionally, it has been found that neutrophils are abundant in chronic wounds (Diegelmann 2003).

The human lung is a well-aerated site, however, in some cases, such as CF, low mucociliary clearance, and thickened mucus form localized depletion of oxygen to permit overgrowth of bacteria in the human lung (Wu et al. 2018). Additionally, invading neutrophils and bacteria consume oxygen resulting in anoxic pockets within the infected mucosa (Downey et al. 2009; Kolpen et al. 2010; Worlitzsch et al. 2002). Studies also showed the large numbers of anaerobic microorganisms in the airways of patients with CF (Tunney et al. 2008; Guss et al. 2011; Sibley et al. 2011). These factors are under hypoxic situations within the lung mucus layer.

Oxygen concentration is a critical parameter for host healing, neutrophil function and signaling, and microbial persistence in biofilm infection (Wu et al. 2018). In a study, Wu et al. (2018) evaluated the fundamental activity of local oxygen concentration in bacterial biofilm infection etiology. Bacteria and host cells consume oxygen, modulate oxygen transport, and actively react to oxygen, causing interactions (Jesaitis et al. 2003; Campbell et al. 2014). They survived the reaction–diffusion interactions during the oxygen concentration gradients (Wu et al. 2018). The Wu et al. (2018) findings showed the co-consumption of oxygen by both host neutrophils and biofilm embedded bacteria that enhance the development of hypoxic situations in the proximity of a biofilm infection. It has been found that these diminished hypoxic levels of oxygen result in diminishing the neutrophil killing ability (Jesaitis et al. 2003; Mandell 1974) because this ability is mediated by the formation of ROS from oxygen Wu et al. (2018). Also, suppose this hypoxic situation continues, and neutrophils will be impaired in their ability to produce ROS like hypochlorous acid and hydrogen peroxide, which causes impairment of the neutrophil's ability to eliminate and control biofilm infection Wu et al. (2018).

#### PMNs

The *P. aeruginosa's* existence in host airways is along with infiltration of PMNs induced by chemoattractant molecules produced by airway epithelial cells and recruited leukocytes (Rada 2017). In this regard, Interleukin-8 (IL-8) could be produced by PMNs, epithelial cells, as well as macrophages and is one of the most potent recruiting chemokines for PMNs that is recognized by chemokine receptors such as C–X–C motif chemokine receptor 1 (CXCR1) and CXCR2 (Rada 2017; Guan et al. 2016). It has been found that IL-8 levels in the airways of CF patients are increased and mediated to lung damage (Colombo et al. 2005; Kim et al. 2006). Also, it has been found that increased IL-8 levels mediated to *P. aeruginosa* infection and detected in the exhaled breath condensate of CF patients, and it was increased in infected patients (Zoumot and Wilson 2010; Bodini et al. 2005). In CF patients, macrophages and PMNs are the primary sources of IL-8 in response to both bacterial lipopolysaccharides (LPS) and host stimuli such as IL-1β and TNF-α (Conese et al. 2003). In this regard, it remains to be further characterize whether the increased level of IL-1β in CF patients is because of intrinsic NF-kB stimulation. However, CF macrophages have an intact inflammasome ability for IL-1β production in response to *P. aeruginosa* (Tang et al. 2012). Although PMNs produce less IL-1β compared to macrophages on a per cell basis, because of the higher total value of PMNs in CF airways, these cells can be a relevant source of IL-1β (Bakele et al. 2014). The lipid LTB4 is one of the most potent factors stimulating PMN chemotaxis and is an arachidonic acid metabolic pathway end-product and released by leukocytes as PMNs (Rada 2017). It has been found that leukotrienes exist in the sputum of CF patients and, also, PMNs from CF have diminished chemotaxis in reacting to LTB4 than PMNs from non-CF patients (Rada 2017; Lawrence and Sorrelli 1992). Also, LTB4 exists in the exhaled breath of CF patients and is increased following the infection of *P. aeruginosa* (Bodini et al. 2005).

PMNs from the airways of CF patients are different from those found in the blood of the same CF patients (Houston et al. 2013; Laval et al. 2013; Makam et al. 2009). PMNs in the CF airways of patients have increased the generation of specific metabolic and stress pathways such as CD39, CD114, C–X–C motif chemokine receptor 4 (CXCR4), as well as Receptor for advanced glycation endproducts (RAGE) (Makam et al. 2009). In this regard, it has been noted that a shift in the formation of their nutrient transporters influences the inorganic phosphate and glucose transports that are part of their adaptation to the airway of CF patients (Laval et al. 2013). PMNs from the CF airways have a diminished respiratory burst and changed TLRs' formation (Rada 2017). Although these cells in the site of CF airways inflammation and infection are well-documented, our knowledge about their metabolic changes is limited at this point.

Of note, about the anaerobic situation in the mucus of CF patients, Kolpin et al. (2010) hypothesized that the respiratory burst of the PMNs involves oxygen consumption. The interactions between PMNs and microorganisms can enhance oxygen consumption because of extra respiration during phagocytosis by the respiratory burst resulting from the electron reduction of oxygen by NADPH oxidase to O-2 (Baldridge and Gerard 1933; Babior et al. 1976). Kolpen et al. (2010) surveyed the respiratory burst ability to deplete oxygen. They provided a simple reaction chamber for real-time evaluation of oxygen concentration during *P. aeruginosa* phagocytosis PMNs. The respiratory burst was provided from aerobic respiration via respiratory chain blocking with potassium cyanide which causes the respiratory burst resistance to stop of the aerobic respiration in PMNs (Aj and Ml 1959). Kolpin et al. (Kolpen et al. 2010) found that CF patients' sputum includes PMNs with an active consumption of oxygen for the formation of O-2 which show that the respiratory burst is continues and trigger accelerated oxygen depletion due to the production of O-2 in chronically infected CF patients.

#### Macrophage

The Warburg effect is now understood as one of the critical features of M1 macrophages, which is essential for increasing the carbon flux in the PPP and for the formation of precursor molecules for anabolic processes and the formation of ROS (O’Neill and Pearce 2016; Torres et al. 2016). Macrophages are exposed to variety of signals, including cytokines of T helper and B lymphocyte, microbes and the host products. Interaction between macrophage and T and B lymphocytes has boosted host immunity and could increase the level of antimicrobial protection (Martinez et al. 2014). The role of IFN-γ in cellular immunity against intracellular infections and the role of IL-4 in parasitic infection leads to the concept of M1 and M2 macrophages, and therefore to a broader range of immune system reactions (Martinez et al. 2014). Macrophages use OxPhos and FAO to exert their anti-inflammatory role, and the metabolic switch is carried out by global changes in gene expression in the cell (Yamada and Kielian 2019). M1 macrophages produce a highly active phosphor fructokinase two isoforms, ubiquitous phosphor fructokinase 2, and downregulate the TCA cycle enzymes that can facilitate the intracellular accumulation of glucose, citrate as well as succinate (Yamada and Kielian 2019).

Additionally, M1 macrophages generate nitric oxide via the up-regulation of iNOS, which directly inhibits OxPhos (Yamada and Kielian 2019). M2 macrophages express the less active fructokinase two phosphorus isoform, phosphate fructokinase B1 and upregulate CD36 to facilitate the uptake of triglycerides for fuel in the TCA cycle (Yamada and Kielian 2019; Feingold et al. 2012). Macrophages in solid tumors undergo metabolic changes via changes in the availability of nutrients, oxygen, and metabolites that coincide with the shifts in inflammatory state (Overmeire et al. 2016). Similarly, the biofilm of S. aureus produces gradients of the proton, nutrients, and oxygen that can impact macrophages' metabolism and subsequently their inflammatory state (Lone et al. 2015; Savijoki et al. 2020).

During their interaction with *P. aeruginosa*, macrophages and neutrophils generate ROS by NADPH oxidase to kill this bacterium (Genestet et al. 2014). Besides, it has been shown that immune reactions are modulated by the indoleamine 2,3-dioxygenase (IDO) of immune cells forming kynurenine via tryptophan (Genestet et al. 2014). Additionally, IDO triggers immunological tolerance via several processes, which have crucial activities in immunological phenomena like allograft acceptance, tumor camouflage, and maternofetal tolerance (Genestet et al. 2014). Furthermore, the IDO stimulation seems to be an essential mechanism for the Human immunodeficiency virus (HIV) to escape from the human immune system (Boasso et al. 2007). More importantly, the kynurenine pathway plays a role in the immune reactions during CF because it has demonstrated that deficiency of IDO activity in CF, forming an imbalance of Th17/Treg that can be recovered by administration of kynurenine metabolite (Iannitti et al. 2013). Taken together, these findings show that IDO has a critical activity in homeostasis of immune reactions to *P. aeruginosa* during CF.

Currently, it has been found that *P. aeruginosa* strains utilize the kynurenine pathway for catabolizing tryptophan (Genestet et al. 2014). Additionally, some host cells generate kynurenine, which is well- defined for modulating of the immune system homeostasis (Genestet et al. 2014). In a study, Genestet et al. (2014) found that *P. aeruginosa* strains isolated from CF patients generate a high kynurenine rate. Furthermore, in *P. aeruginosa*, kynA gene, a transcriptional stimulation (involved in the kynurenine pathway) was found following contact of *P. aeruginosa* with immune cells such as neutrophils (Genestet et al. 2014). Besides, Genestet et al. (Genestet et al. 2014) via neutrophils and *P. aeruginosa* producing no (ΔkynA) and high rate of kynurenine (ΔkynU) co-cultures showed that kynurenine enhances the survival of this bacterium.

Interestingly, an increase in the rate of kynurenine hampers the production of ROS by stimulated neutrophils. Genestet et al. (2014), via a ROS-forming system, found that kynurenine scavenges hydrogen peroxide, as well as superoxide that occurs usually following bacterial induction, particularly in the phagosome. In summary, the kynurenine pathway enables *P. aeruginosa* to circumvent the immune reaction by scavenging ROS formation by neutrophils. Overall, these results prove the influence of kynurenine produced by bacteria on neutrophils' killing activity via ROS scavenging.

It has been found that macrophages related to the biofilm of *S. aureus* are polarized to an M2 phenotype. The adoptive transfer of the M1 phenotype results in attenuated biofilm burden, proposing the critical activity of macrophage inflammatory responses during biofilm infection of *S. aureus* (Yamada et al. 2020). The inflammatory status of leukocytes is associated with their metabolic situation. In this regard, in a study by Yamada et al. (2020), monocytes associated with biofilm show a metabolic favoring to OxPhos and less aerobic glycolysis for prompting their anti-inflammatory role that causes persistence of *S. aureus* biofilm. To change monocyte and reprogram immune cells' metabolism to a pro-inflammatory situation, they used a nanoparticle approach to deliver the oligomycin, an inhibitor of OxPhos, to monocyte cells (Yamada et al. 2020). Yamada et al. (2020) targeted the nanoparticle uptake to monocytes by conjugating with tuftsin and incorporated a fluorochrome to monitor nanoparticles' distribution, resulting in preferentially internalized monocytes rather than MDSCs and PMNs (Yamada et al. 2020). They delivered an oligomycin nanoparticle an in vivo* S. aureus* prosthetic joint infection model to monocytes, which notably diminished the biofilm burden by changing the monocyte metabolism and enhancing the pro-inflammatory state of infiltrating monocytes (Yamada et al. 2020). Yamada et al. (2020) showed that oligomycin injection alone had no influence on the metabolism of monocyte cells and biofilm burden and interestingly found that specific intracellular transfer of oligomycin is crucial for reprogramming the metabolic activity of monocyte cells (Yamada et al. 2020). Of note, metabolic reprogramming of monocyte cells with oligomycin nanoparticles combined with antibiotics causes the elimination of established *S. aureus* biofilm (Yamada et al. 2020). These results show that the metabolic reprogramming monocytes associated with biofilm can propose a novel therapeutic method for prosthetic joint infection.

Additionally, in a study, Hanke et al. (2013) administered the macrophage-activating peptide EP67 (Tyr-Ser-Phe-Lys-Asp-Met-Pro (N-methyl Leu)-d-Ala-Arg) to enhance the clearance of bacterial biofilm by performing a proinflammatory status. EP67 is an agonist for the selective reaction of the C5a receptor (C5aR/CD88) and preferentially triggers proinflammatory mediators from CD88+ macrophages (Hanke et al. 2013). Hanke et al. (2013) found that targeting macrophages' properties with EP67 suppresses the biofilm formation of *S. aureus*, proposing a novel therapeutic approach for this kind of infection. The therapeutic ability of activated macrophages shows that primary treatment with M1 macrophages significantly reduced the biofilm growth of *S. aureus* (preventive effect). Besides, activated macrophages effectively diminished the *S. aureus* burdens in established biofilm (degradative effect) (Hanke et al. 2013). The cytokine and chemokine condition produced after M1 macrophages reflects products derived from T lymphocytes like IL-17 and IFN-γ and macrophages like IL-1, C–X–C motif chemokine ligand 9 (CXCL9), and C–C motif chemokine ligand 5 (CCL5), showing the coordinate role of these immune cells (Hanke et al. 2013). This situation was only crucial in the primary glance with M1 macrophages, and the other functional influence of these factors on biofilm burdens must be evaluated (Hanke et al. 2013). The administration of EP67 can overcome the immune dysfunction during biofilm infection, as EP67 offers the correct stimulation signals to CD88+ macrophages to produce a powerful bactericidal reaction during biofilm infection (Hanke et al. 2013). In summary, these results show that immune cell-based therapy via activated macrophages can open the door to overcome the current problems mediated by biofilm infections.

**Fig. 7:**
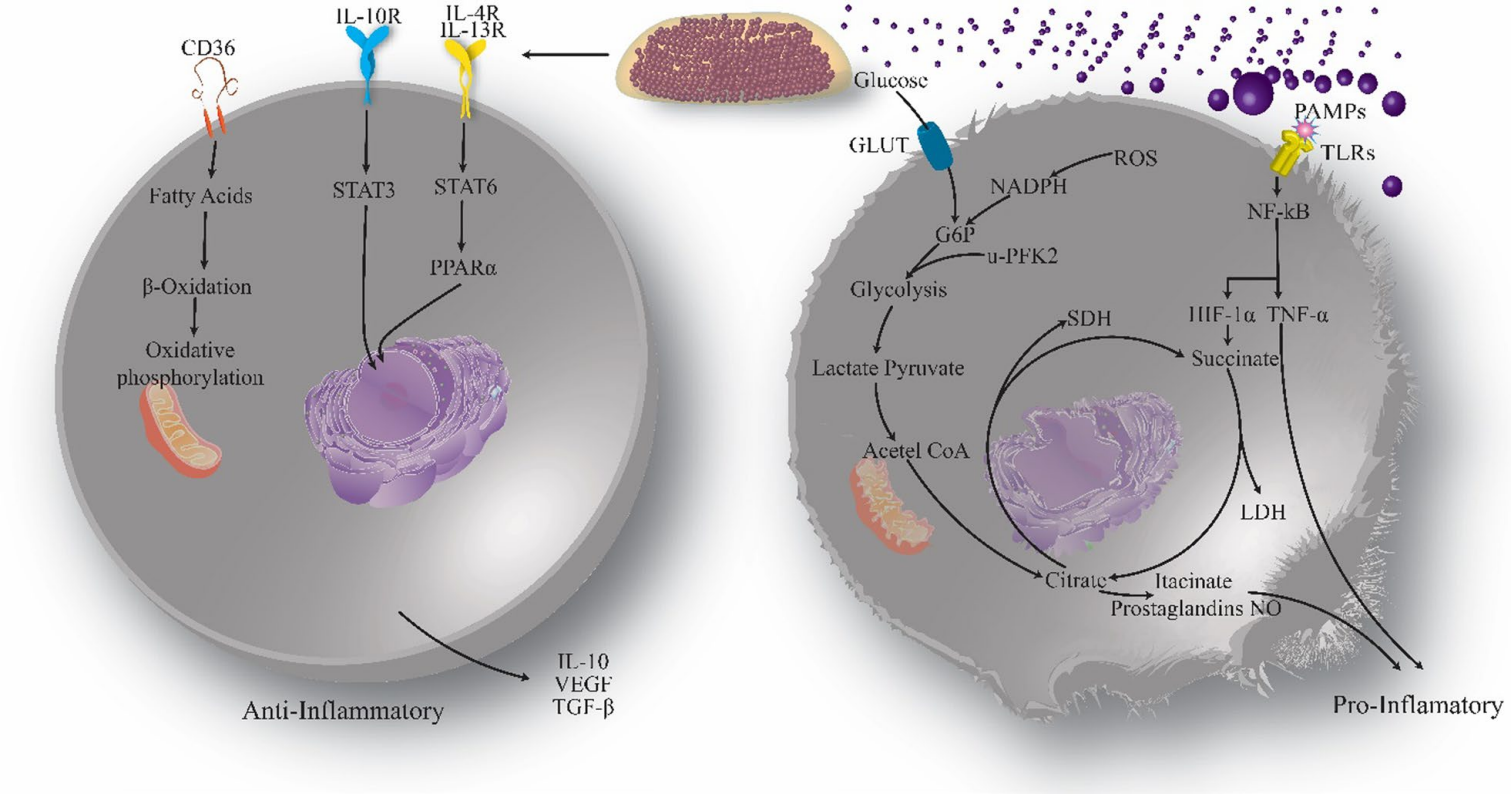
Macrophages and their metabolic changes can change their inflammatory status to a biofilm infection. In planktonic infections, macrophages encounter pathogen-associated molecular patterns (PAMPs) that favor aerobic glycolysis to provide tricarboxylic acid (TCA) cycle intermediates required for proinflammatory effector mechanisms. In contrast, macrophages are polarized towards an anti-inflammatory state in biofilm infections. Although most of the factors involved in this process are unknown, it is predicted that biofilm infections will bias macrophages towards OXPHOS and several receptors such as IL-4R, IL-13R, IL-10R, and CD36 which are associated with anti-inflammatory cytokines (e.g., IL-10 and TGF-β) might be involved. The metabolic gradients of nutrients and oxygen present in the tissue microenvironment also influence macrophages' anti- versus pro-inflammatory states (Yamada and Kielian 2019). G6P, glucose-6-phosphate; IDH, isocitrate dehydrogenase; iNOS, inducible nitric oxide synthase; PPP, pentose phosphate pathway; SDH, succinate dehydrogenase

Most importantly, currently, it has been found that metabolites like succinate and itaconate could be utilized as signaling molecules, metabolic intermediates, and triggers for a functional phenotypic change of macrophages (Tannahill et al. 2013; Lampropoulou et al. 2016, 2021). Previous works have found that M1 and M2 macrophages show distinct metabolite profiles such as fatty acid utilization, OxPhos, and glycolysis (Fuchs et al. 2020). Nevertheless, many evaluations are demanded to characterise macrophages' immunometabolic properties. The potential metabolic and functional differences mediated to macrophages react to invasive bacterial pathogens, particularly concerning differential phenotypic reactions of macrophages to planktonic versus biofilm growth mode (Fuchs et al. 2020). In a study, Fuchs et al. (2020) aimed to evaluate the metabolic influences of planktonic and biofilm *P. aeruginosa* on primary monocyte-derived resting (M0) macrophages via in vitro macrophage differentiation and exposure schemes (CD14+ magnetic-activated cell sorting), 1D 1H NMR metabolomics, as well as metabolic profiling analysis. In their study, M0 macrophages were differentiated from primary monocytes via macrophage colony-stimulating factor (M-CSF) before activation with planktonic and biofilm *P. aeruginosa* (Fuchs et al. 2020). After exposure, both macrophage intra- and extracellular metabolites were isolated, and 1D 1H NMR spectra were obtained, as well as metabolite profiling of these NMR spectra was calculated (Fuchs et al. 2020). Findings of Fuchs et al. (2020) proposed significant changes in metabolite profiles and show some metabolic pathways that are differentially increased in planktonic- and biofilm-exposed macrophages such as purine biosynthesis glycolysis, branched-chain amino acid catabolism, inositol phosphate metabolism, as well as glycerol metabolism (Fuchs et al. 2020). Besides, these metabolic patterns showed that biofilm-exposed macrophages have a hyper-inflammatory metabolic profile, diminished glycerol metabolism, and increased catabolism of amino acids and lactate compared to the planktonic exposed macrophages (Fig. 7) (Fuchs et al. 2020). Collectedly, this study proposes novel results on the metabolic modulation of macrophages following exposure to bacteria and biofilm and opens the road to additional knowledge in the field of immunometabolism.

OxPhos and glycolysis are entirely coupled and act as the molecular exchange system; for example, glycolysis occurs in the host cytoplasm and forms pyruvate, which is the fuel for OxPhos. Nevertheless, as glycolysis increases, concomitant increases in the PPP are generally observed with ribose phosphate and NADPH that are needed for cellular functions, biosynthesis, and cell division (O’Neill and Pearce 2016; Loftus and Finlay 2016). This critical aspect of the immune system regulates the cell life span, influencing the population of memory lymphocytes and differentiated leukocytes against bacterial infections. Some research suggests that oxidative metabolism of phosphorylation promotes the survival of immune cells. For example, M2 macrophage (use OxPhos) has a longer lifespan, whereas M1 macrophage, which uses glycolysis, is short-lived (Loftus and Finlay 2016). These phenomena are most commonly observed in memory T lymphocytes, where T lymphocytes do not oxidize glucose at rest but instead use FAO (Wang and Green 2012).

Regarding biofilms, it was shown that macrophages that reached biofilm infection of *S. aureus* show M2 profiles (Heim et al. 2014; Heim et al. 2015). Cytokine levels decreased during biofilm device infection of mice deficient on MyD88. It was thought that other cytokines triggered during glycolysis could help maintain biofilm viability by lytic toxins of biofilm bacteria and apoptosis of effector cells. Although cytokine-induced apoptosis of macrophages and neutrophils occurs during planktonic infections, biofilm infections' static and chronic nature enhances cytokine accumulation, as seen with *S. aureus* biofilm infection (Heim et al. 2015; Heim et al. 2018).

## Immunometabolite itaconate

One of the most essential components of the immune response to infection is the production of host metabolites, which control inflammation and the survival of pathogens (Tomlinson and Riquelme 2021). The researchers discovered that *S. aureus* stimulates the formation of the immunomodulatory metabolite itaconate in airway immune cells via increasing mitochondrial oxidative stress in a study were performed by Tomlinson et al. (2021). Itaconate decreased *S. aureus* glycolysis and proliferation to address these effects while increasing carbon influx via bacterial metabolic pathways that contribute to biofilm development (Tomlinson et al. 2021). Researchers found that itaconate-induced metabolic alterations were repeated in a longitudinal number of clinical isolates from a case with persistent Staphylococcal lung diseases, indicating that host immunometabolism plays a role in promoting bacterial survival throughout long-term Staphylococcal lung diseases. TCA cycle abnormalities in M1 macrophages cause deposition of TCA cycle intermediates, particularly citrate and succinate (Yamada and Kielian 2019). Succinate deposition increases HIF-1α stability, epigenetic alterations, and IL-1 synthesis by suppressing α-ketoglutarate-dependent enzymes (Loftus and Finlay 2016). Itaconate is an antimicrobial metabolite that suppresses planktonic Salmonella enterica and *Mycobacterium tuberculosis* development and may be generated from citrate (Loftus and Finlay 2016). Nevertheless, itaconate's antimicrobial impacts on biofilm infections have still not been investigated. Moreover, citrate is employed as a substrate for the formation of nitric oxide, reactive oxygen species, lipids, and prostaglandins. Because, macrophages are anti-inflammatory in nature during biofilm infections, they are believed to rely predominantly on OxPhos metabolism for their energy needs. Nevertheless, as a widespread survival mechanism, some bacteria, notably *Yersinia Pestis* and *P. aeruginosa*, disrupt itaconate by producing three enzymes: itaconyl-CoA hydratase (S) citramalyl-CoA lyase, and itaconate coenzyme A (CoA) transferase (Sasikaran et al. 2014). Moreover, *P. aeruginosa* uses itaconate as a carbon source, enabling it to form biofilm in the lungs of CF patients. In contrast to other opportunistic microbes, Riquelme et al. (2020) discovered that P. aeruginosa changes its metabolic and immunomodulatory features in reaction to itaconate, a common host-derived immunometabolite in the affected lung. Itaconate promotes bacterial membrane distress, which causes LPS to be downregulated and EPS to be upregulated (Riquelme et al. 2020). *P. aeruginosa* that has evolved to itaconate accumulates lptD mutations, which promote itaconate absorption and biofilm production. EPS could stimulate the synthesis of itaconate by myeloid cells, both systemically and in the airway system, skewing the host immune immunity to one allowed of persistent infection (Riquelme et al. 2020).

Current findings have also shown that itaconate plays a critical function in the immunometabolism of cancers (Lin et al. 2021). Itaconate has been shown to have anti-cancer properties in patients with colorectal cancer associated with colitis (Wang et al. 2020). IL-1β and CCL2 release were hindered in intestinal epithelial cells after administering dimethyl itaconate, which also diminished the penetration of M1 macrophages into the tumor microenvironment, resulting in a reduction in the elevated inflammatory processes associated with ulcerative colitis. Itaconate additionally reduced the risk of colitis-associated colorectal cancer progression by decreasing the development of cytotoxic T cells and subsequent infiltration of MDSCs. According to previous research, high levels of itaconate have been linked to cancer development and poor prognosis in some tumor forms (Weiss et al. 2018). The tumor milieu is dominated by tumor-associated macrophages, which may release IL-10 and growth factors that promote tumor development, aggression, and metastasis as one of the most essential elements (Vitale et al. 2019; Noy and Pollard 2014). In B16 melanoma and ID8 ovarian cancer, intraperitoneal tissue-resident macrophages were shown to upregulate Irg1 expression and raise itaconate concentrations (Weiss et al. 2018). It has been shown that itaconate increases OxPhos, resulting in ROS formation, in turn activating the mitogen-activated protein kinase (MAPK).

In contrast, when the transformation to itaconate in Irg1−/− mice was blocked, a substantial decrease in tumor volume was found in mice (Weiss et al. 2018). Itaconate also increased the production of ROS in intraperitoneal tissue-resident macrophages mediated by OxPhos, which was fueled mainly by the NADH cycle, a succinate dehydrogenase-independent process, as previously reported (Basit et al. 2018). Irg1 was produced in high concentrations in monocytes separated from the ascetic fluid of ovarian cancer patients. Similarly, other researchers noted that Irg1 performed an oncogene-like function in glioma, which resulted in an unfavorable prognosis for the patients (Pan et al. 2014). In order to thoroughly understand the immunomodulatory properties of itaconate on various kinds of cancers, further studiew are required.

Nowadays, it has been well defined that the biofilm in the wound is the main factor in the failure of therapy and expansion of non‐healing, chronic human wounds. In a study, Ammons et al. (2017) surveyed how metabolite competition and condition between macrophages and biofilm produced by *P. aeruginosa* promotes M1 polarization of macrophages and skews macrophages into an M2 phenotype. Ammons et al. (2017), via metabolomics by 1D 1H Nuclear magnetic resonance (NMR) along with immunologic evaluations, showed substantial overlap between the metabolic profile driving macrophage polarization either into the M1 phenotype or the M2 phenotype and showed that the *P. aeruginosa* biofilm included crucial metabolic pathways that modulate immunomodulation in macrophages like shifts between the PPP and TCA cycle and amino acid metabolism Rada (2017). They found that exposure of non‐polarized macrophages to small metabolites from planktonic- and biofilm mode of *P. aeruginosa* resulted in distinct metabolic pathways involved in macrophage immunomodulation including increased glycolysis, uncoupling of the TCA cycle, and selective uptake and metabolism of amino acids Rada (2017). Additionally, Ammons et al. (2017) used biofilm‐targeted therapeutics to handle macrophage polarization immunomodulation during biofilm infection to enhance a shift from an M1 phenotype to an M2 phenotype in un-activated macrophages. Taken together, they found that *P. aeruginosa* biofilms relevant to specific pathological influences toward macrophages via coordinated metabolic interplays, which cause immune polarization and can involve deviation from the usual healing process of wound and expansion of a chronic host wound Rada (2017). These results can lead to new methods for treatment and prevention of non‐healing wounds, such as developing treatment protocols using immune metabolites.

## Immunometabolism as a novel therapeutic approach

The idea that metabolic shifts modify the phenotype and role of immune cells in the tumor microenvironment is supported and assumed to at least partially lead to acquired resistance to tumor immunotherapy (Guo et al. 2019). The critical objective of immunotherapy is to counteract the suppression of the immune system by efficiently mobilizing cancer-specific T-effector cells and producing T-memory cells that enable long-term immune-mediated removal or regulation of tumors (Guo et al. 2019). Because metabolic programming encourages the production and functionality of immune cells, the use of metabolic medications may provide new strategies to promote tumor immunotherapy (Guo et al. 2019). Evidence is emerging that metabolic control, e.g., repression of cholesterol esterification, suppressing adenosine monophosphate transfer, and targeting imbalanced lipid aggregation in tumor-infiltration dendritic cells, can be used to improve or normalize immune functions for improved cancer care (Yang et al. 2016; Allard et al. 2013; Cubillos-Ruiz et al. 2015; Herber et al. 2010). A complete list of immunometabolism-based therapies for cancer is provided in previous studies (Beezhold and Byersdorfer 2018, Mockler et al. 2014, Singer et al. 2018; Guo et al. 2019; Mazumdar et al. 2020).

Immune cell activation and sufficient differentiation are crucial in various infections (Ng et al. 2013, 2002). Characterizing different signaling pathways and transcription patterns in immune cell subsets can lead to the identification and offer of new targets for improving the treatment of biofilm infections. It has also been revealed that the reprogramming of cellular metabolic pathways has a vital role in triggering and controlling immune cell reactions (Pearce and Pearce 2013). Accordingly, more attention has been given to interrogating and manipulating human immune cells' metabolic reactions to develop new therapeutic methods in infectious diseases. Once immune cells are activated, metabolic rescheduling and nutrient uptake changes are essential for biosynthetic pathways in terms of cell division and function (Pearce and Pearce 2013). This mode is defined by increased glutamine metabolism, glycolytic rate, PPP, and the formation of nucleic acids, proteins, and lipids (Polat 2016). For example, the metabolic programs used by different T cell subgroups appear to have distinct features that are essential to support the strengthening and function of their lines (Slack et al. 2015).

In the case of biofilm, *S. aureus* has been shown to stimulate the development of MDSCs strongly, and MDSCs cause severe infections (Ost et al. 2016). Nevertheless, MDSCs were mediated to an enhanced outcome like *P. aeruginosa* in CF patients (Ost et al. 2016). A powerful therapeutic approach can target MDSCs in *S. aureus* biofilm implant infections. Further knowledge remains on how to translate these results into therapeutic approaches. For example, the tyrosine-kinase inhibitors, e.g., Sunitinib, interfere with STAT3 signaling and reduce MDSCs (Ost et al. 2016). The same outcome could also be achieved using an active metabolite of vitamin (all-trans-retinoic acid (ATRA)) (Ost et al. 2016).

This review aimed to provide an overview of the emergence of immunometabolism in infectious diseases, especially biofilm infections. Therefore, we have provided examples in various studies in which immune cell metabolism is involved in immune responses to bacterial infections, especially biofilm infections. The dynamic nature of metabolic programming among immune cells is closely related to their flexibility and function. The metabolism pathways have recently been targeted for some autoimmune and inflammatory diseases (for example, calcineurin inhibitors (cyclosporine and FK506 suppress effector and regulator cells), and metabolic therapy approaches can offer a promising field of research into the treatment and control of infectious diseases (Shen et al. 2016; Harrison et al. 2007; Huang and Perl 2018, 2019). A growing body of evidence shows how metabolism inhibitors (glycolysis, glutamine metabolism, and fatty acid oxidation) can regulate human immune responses and cure immune-mediated pathogenesis. For example, drugs that target IL-1β exhibited promising results in clinical trials (Ayres 2020). Targeting immune and metabolic regulators can represent a promising novel approach for curing and preventing bacterial infections. Various studies have examined metabolic alterations during infection and highlighted the importance of metabolism in host defense to pathogens.

## Conclusion

Biofilm plays a crucial role in the prognosis and progression of various bacterial infections that are often associated with chronic states and cause long-term problems for patients. Mechanisms of stability and resistance of biofilm bacteria present unique and evolving challenges to patients and physicians. For this reason, biofilm research is receiving more attention. Interestingly, biofilm infections have been shown to modify the host's peripheral environment. Most importantly, it has been shown that biofilm infections can change the inflammatory immune reactions, as evidenced by macrophage polarization and recruitment of MDSCs, to anti-inflammatory modes. Overall, these findings show that biofilm infections can change immune cells' metabolism, affecting the development and activation of immune reactions (Table 3). Innovative therapeutic agents in infections are crucial to overcoming biofilm-associated infections' severe complications. We suggest that more attention should be given to interrogating and manipulating the metabolic reactions in immune cells to develop new therapeutic methods in biofilm infections. Besides, combining bacteriotherapy approaches with chemotherapy can help defeat tumor heterogeneity accompanied by malignancy, drug resistance, and metastasis.

## Data Availability

Not applicable.

## Abbreviations

EPS: Extracellular polymeric substances
MDSCs: Myeloid-derived suppressor cells
LPS: Lipopolysaccharides
OxPhos: Oxidative phosphorylation
IL-17: Interleukin-17
ETBF: Enterotoxigenic Bacteroides fragilis
IBD: Inflammatory bowel diseases
IL-6: Interleukin 6
STAT3: Signal transducer and activator of transcription 3
OMV: Outer membrane vesicles
PCR: polymerase chain reaction
IgG: Immunoglobulin G
OMP: Outer membrane protein
Cag: Cytotoxin-associated gene
PAI: Pathogenicity island
TCA: Ricarboxylic acid
NADPH: Nicotinamide adenine dinucleotide phosphate
ATP: Adenosine Triphosphate
FAD: Flavin adenine dinucleotide
LAT1: L-Type Amino Acid Transporter
APCs: Antigen-presenting cells
CTL: Cytotoxic T lymphocytes
IL-2: Interleukin-2
IFN-γ: Interferon-γ
IL-4: Interleukin-4
IL-13: Interleukin-13
IL-10: Interleukin-10
IL-23: Interleukin-23
TNF-α: Tumor necrosis factor-α
IgG2a: Immunoglobulin G2a
TCR: T-cell receptor
IL-12: Interleukin 12
IL-18: Interleukin-18
IL-5: Interleukin-5
IgE: Immunoglobulin E
TGF-β: Transformation of the growth factor-β
IgM: Immunoglobulin M
IgA: Immunoglobulin A
Tregs: Regulatory T cells
FOXP3: Forkhead box protein P3
CTLA4: Cytotoxic T-lymphocyte antigen 4
M1: Pro-inflammatory macrophages
M2: Anti-inflammatory macrophages
RBPJ: Recombination signal binding protein for the immunoglobulin kappa J region
ARG1: Arginase 1
IRF4: Interferon regulatory factor 4
SOCS3: Cytokine signaling suppressor 3
CCL1: Chemokine ligand 1
IL-1: Interleukin-1
PMNs: Polymorphonuclear leukocytes
ROS: Reactive oxygen species
NET: Neutrophil extracellular trap
QS: Quorum sensing
AHL: N-acyl homoserine lactone
CXCL2: C–X–C Motif Chemokine Ligand 2
INOS: Inducible nitric oxide synthase
TLRs: Toll-like receptors
GM-CSF: Granulocyte–macrophage colony-stimulating factor
VEGF: Vascular endothelial growth factor
G-CSF: Granulocyte-colony stimulating factor
FnBP: Fibronectin-binding protein
Cas8: Caspase 8
SCV: Small colony variant
IL-1R: IL-1 receptors
MyD88: Myeloid differentiation primary response 88
CXCL1: Chemokine (C–X–C motif) ligand 1
GAPDH: Glyceraldehyde-3-phosphate dehydrogenase
NFAT: Nuclear factor of activated T cells
PI3K: Phosphatidylinositol 3-kinase
AKT: Protein kinase B
MTORC1: Mammalian target of rapamycin complex 1
CNS2: Conserved non-coding sequence 2
PPP: Pentose phosphate pathway
TAMs: Tumor-associated macrophages
HIF1α: Hypoxia-inducible factor 1 alpha
CF: Cystic fibrosis
IL-8: Interleukin 8
CXCR1: C–X–C motif chemokine receptor 1
CXCR2: C–X–C motif chemokine receptor 2
LTB4: Lipid leukotriene B4
CXCR4: C–X–C motif chemokine receptor 4
RAGE: Receptor for Advanced Glycation Endproducts
HIV: Human Immunodeficiency Virus
NMR: Nuclear Magnetic Resonance
M-CSF: Macrophage colony-stimulating factor
